# Disentangling Enhanced Diffusion and Ballistic Motion of Excitons Coupled to Bloch Surface Waves with Molecular Dynamics Simulations

**DOI:** 10.1021/acs.jpclett.5c01391

**Published:** 2025-06-24

**Authors:** Ilia Sokolovskii, Yunyi Luo, Gerrit Groenhof

**Affiliations:** Nanoscience Center and Department of Chemistry, 205518 University of Jyväskylä, P.O. Box 35, 40014 Jyväskylä, Finland

## Abstract

Placing an organic material on top of a Bragg mirror can significantly enhance the exciton transport. Such enhancement has been attributed to strong coupling between the evanescent Bloch surface waves (BSW) on the mirror and the excitons in the material. In this regime, the BSW and excitons hybridize into Bloch surface wave polaritons (BSWP), new quasiparticles with both photonic and excitonic character. While recent experiments unveiled a mixed nature of the enhanced transport, the role of the material degrees of freedom in this process remains unclear. To clarify their role, we performed atomistic molecular dynamics simulations of an ensemble of methylene blue dye molecules strongly coupled to a BSW. The simulations reveal a correlation between the photonic content of the BSWP and the nature of the transport. In line with the experiment, we find ballistic motion for polaritons with high photonic character and enhanced diffusion if the photonic content is low. Our simulations furthermore suggest that the diffusion is due to (*i*) excitation energy disorder of the molecules and (*ii*) thermally activated vibrations that drive population transfer between the stationary dark states and mobile bright polaritonic states. Importantly, the transition to diffusion at a low photonic content cannot be fully captured by static models of polaritons, underscoring the importance of dynamical effects (thermal disorder and nonadiabatic coupling) on transport of organic polaritons.

## Graphical abstract

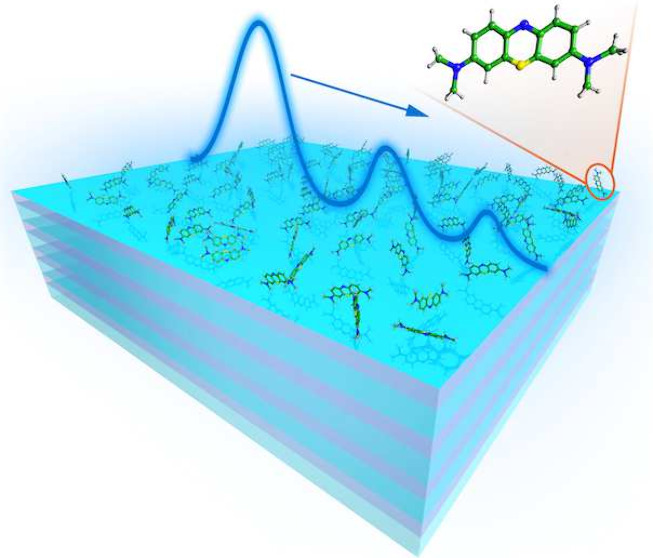

The propagation of Frenkel excitons in organic materials is a diffusion process in which the excitons hop between adjacent molecules via dipole–dipole coupling (Förster mechanism)^1^ or wave function overlap (Dexter mechanism).^2^ Because the efficiency of these mechanisms depends on the intermolecular separation and orientation, structural disorder has a negative impact on exciton transfer, resulting in a limited diffusion length of Frenkel excitons, typically below 10 nm.^3^

The propagation distance of excitons can be increased by placing organic molecules in a confined electromagnetic field, as found inside an optical microcavity or near a plasmonic surface.^4^ There, excitons can strongly interact with the confined light modes of the optical structure. If the strength of this interaction exceeds the rates associated with losses in the system, the excitons and confined light modes hybridize into polaritons,^5,6^ which inherit the properties of both constituents of the interaction, including dispersion and hence group velocity. This allows for a long-range propagation of polaritons beyond the diffusion length of Frenkel excitons, as has been demonstrated in various types of optical structures.^7–17^

Theory has provided important insights into the mechanisms by which strong coupling can enhance exciton transport,^18–30^ but the description of the material in these works has been limited to two-level systems. To go beyond such simplified model systems and also consider the vibrational degrees of freedom, several models have been proposed,^15,16,31–33^ including a simulation model based on multiscale molecular dynamics,^34,35^ in which the structural details of the material are explicitly included. With such simulations, we could demonstrate that polariton wave packets propagate in a diffusive manner due to reversible population exchanges between stationary dark states and propagating bright states, which get populated along the whole lower polariton branch.^36,37^ While experiment^38^ and theory^28,39,40^ suggest that the coherence of polaritons, and hence the propagation distance, increases with their photonic weight, it remains unclear whether propagation of individual wave packets constituting the total polaritonic wave packet, is fully ballistic or might also be diffusive depending on the wave vector at which an individual wave packet is formed.

Recently, this question was addressed in two separate experimental studies.^16,17^ In the first study, conducted by Balasubrahmaniyam et al., spatiotemporal ultrafast pump–probe microscopy was used to track polariton transport in a system of 5,5′,6,6′-tetrachloro-1,1′-diethyl-3,3′-di­(4-sulfobutyl)­benzimidazolocarbocyanine (TDBC) J-aggregates deposited on top of a distributed Bragg reflector (DBR) supporting Bloch surface waves (BSWs).^17^ By probing the differential reflectivity, *ΔR*/*R*, at different angles and energies as a function of time after off-resonant excitation into the J-aggregates, a transition from ballistic propagation with a velocity close to the corresponding polariton group velocity to diffusive propagation with a much lower velocity was observed when the photonic contribution to the polaritonic states decreased. This transition was attributed to a competition between molecular-scale disorder and long-range correlation due to strong coupling, with the former prevailing at small photonic fractions.

In the second study, carried out by Xu et al.,^16^ polariton transport was observed in a variety of inorganic exciton-cavity structures by means of a momentum-resolved ultrafast polariton imaging technique. As in Balasubrahmaniyam et al.,^17^ both a deviation of the propagation speed from the polariton group velocity and a transition from ballistic to diffusive transport was observed as the photonic contribution to the polaritonic states decreased and was attributed to a more intensive scattering by lattice phonons.

Thus, the results of the two experiments agree that polariton transport undergoes a crossover between ballistic and diffusion regimes when the polariton states become more exciton-like. However, in the case of organic molecules, it remains unclear whether this crossover is due to reversible nonadiabatic population transfers between bright and dark states, or whether such a transition can be caused solely by structural molecular disorder. To address this question, we mimic the experiment of Balasubrahmaniyam et al.^17^ by means of multiscale quantum mechanics/molecular mechanics (QM/MM) molecular dynamics (MD) simulations.^34,41^ The results of our simulations suggest that the change in the transport regime is caused by both excitation energy disorder and vibrationally induced nonadiabatic population exchanges between polaritonic and dark states. However, the effect cannot be fully captured by static excitation energy disorder alone, which underscores the importance of molecular vibrations in the transport of organic exciton-polaritons.

Before considering the details of our MD simulations, we briefly discuss the origin of the formation of BSWs in a distributed Bragg reflector (DBR). As illustrated in Figure 1, a DBR is a one-dimensional photonic crystal, a structure in which the electric permittivity varies periodically, i.e., *ε*(*x*) = *ε*(*x* + *A*) with period *A*. Just as the periodicity of ions in an atomic crystal leads to the appearance of allowed bands and band gaps, the periodicity of the permittivity in the DBR leads to the appearance of so-called pass-bands and stop-bands. For an electromagnetic (EM) wave with a frequency inside a stop-band, multiple reflections of this wave from the boundaries between layers with different permittivity result in the occurrence of destructive interference, which makes it impossible for the light to travel through the DBR. However, the introduction of a defect, such as a layer with a thickness or permittivity different from that of the other layers, may allow for localized states to appear in the stop-band.^42^

**Figure 1:**
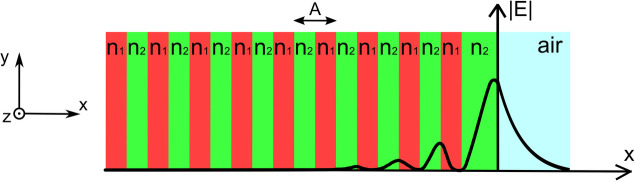
Schematic representation of a distributed Bragg reflector (DBR) supporting a Bloch surface wave (BSW). The DBR consists of alternating layers with different refractive indices, *n*_1_ and *n*_2_, with period *A* along the *x*-axis. Additionally, a surface defect layer with refractive index *n*_2_ and a thickness different from the thicknesses of the other layers is introduced. The electric field strength distribution of the BSW is shown as a black line.

In addition to EM waves propagating within the volume, DBRs support (*i*) surface waves that propagate in both dielectric and air (states in pass-bands above the light line), (*ii*) surface waves that propagate in the dielectric while decaying in air (states in pass-bands below the light line), and (*iii*) states that decay within the dielectric while propagating in air (states in stop-bands above the light line).^43^ The introduction of a surface defect results in the appearance of a fourth type of surface wave, which is a wave localized in both dielectric and air.^44^ This is the Bloch surface wave.

Because BSWs exist within the stop-bands and below the light line,^43^ i.e., at angles larger than the critical angle for total internal reflection, the radiation cannot be emitted into free space, which results in an extremely long lifetime that is significantly higher than for typical Fabry–Pérot microcavities with metallic mirrors, or plasmonic structures. As a consequence, the lifetime of polaritons formed due to strong coupling between excitons and BSWs, can reach several hundreds to thousands of femtoseconds.^8,12,45^ Because the BSW is a surface wave, evanescent in the direction perpendicular to the surface (i.e., *x* in Figure 1), there is no restriction on the in-plane propagation over the surface of the Bragg mirror (i.e., in the *y*- and *z*-directions). Therefore, the electric field distribution of the BSW in the air is defined as

1 $$E\left(x,y,z\right)=E_{0}e^{-\left|K\right|x}e^{i\left(k_{y}y+k_{z}z\right)}$$

with *E*_0_ the amplitude of the electric field at the surface of the DBR (i.e., at *x* = 0) and *K* the complex Bloch wavenumber.^46^ A major advantage of BSW-polaritons over polaritons in Fabry–Pérot cavities is the much higher group velocity of the LP branch. In the BSW structures, the dispersion is close to the light line in free space and the group velocity of the LP branch can approach the speed of light. This results in a tremendous propagation of BSW-polaritons reaching tens to hundreds of micrometers.^8,12,17,47^

To investigate this propagation at an atomic resolution, we perform mean-field QM/MM molecular dynamics simulations of *N* = 1024 methylene blue molecules (MeB) in water coupled to a one-dimensional Bloch Surface Wave (Figure 2) at room temperature. The electronic ground (S_0_) and excited states (S_1_) of MeB are described at the level of density functional theory (DFT)^48^ and time-dependent DFT (TDDFT),^49,50^ respectively, using the Becke97 functional^51^ in combination with the 3-21G basis set,^52^ while the water molecules are modeled with the TIP3P force field.^53^ Here, we choose to model MeB, rather than the TDBC J-aggregates used in the experimens of Balasubrahmaniyam et al.,^17^ as the latter’s complexity makes their simulation in a cavity currently intractable. Nevertheless, in spite of their larger absorption line width, the MeB molecules share the most important features of the J-aggregates, namely, a bright electronic transition and several vibrational modes that are Raman-active.^54^ At the level of TDDFT theory employed in this work, the excitation energies of MeB in water (2.5 eV) are significantly overestimated with respect to experiment (1.9 eV). Such discrepancies have also been noted in previous works,^55,56^ and were attributed to the large difference in the charge density between the electronic ground and excited states, which is notoriously difficult to describe accurately with TDDFT.^57^

**Figure 2:**
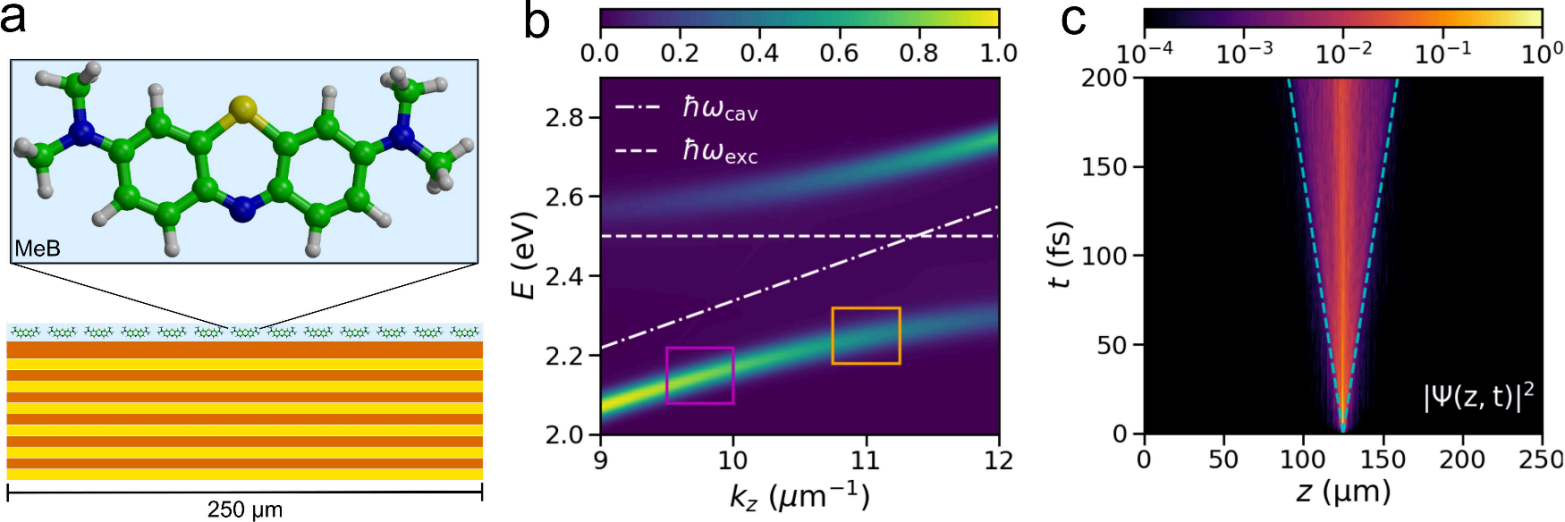
Panel a: Schematic illustration of MeB molecules deposited on top of a DBR. The electronic ground (S_0_) and excited (S_1_) states are calculated at the QM level with density functional theory (DFT) and time-dependent (TD-)­DFT, respectively, by using the B97 functional and the 3-21G basis set. The solvent molecules (water, not shown) are described at the MM level with the TIP3P model. Carbon atoms are shown in green, nitrogen atoms in blue, sulfur atom in yellow, and hydrogen atoms in gray. Panel b: Normalized angle-resolved absorption spectrum of the MeB-Bloch surface wave (BSW) system. The dashed line corresponds to the excitation energy of MeB at 2.50 meV at the TDDFT-B97/3-21G level of theory, and the dashed-dotted line shows the BSW dispersion. The purple and orange rectangles illustrate two wave vector windows, from which the partial wave functions, |Ψ_part_⟩, are extracted and plotted in Figure 3. For clarity, only the positive part of the absorption spectrum between *k*_*z*_ = 9 μm^–1^ and *k*_*z*_= 12 μm^–1^ is shown. Panel c: Space-time map of the probability amplitude of the total wave function |Ψ­(*z, t*)|^2^. The dashed lines indicate the maximum group velocity of the LP branch, υ_LP_^max^ = 172 μm ps^–1^.

Although there is currently no consensus on how many J-aggregates were coupled in the experiments of Balasubrahmaniyam et al.,^17^ this number is most likely larger than the 1024 molecules used in our simulations. Through a systematic variation of the number of molecules we could previously establish that the mechanism of propagation is not very sensitive to the number of molecules included in the MD simulations but that the transport velocity is inversely proportional to that number.^36^ Since here we want to find out how vibrationally mediated nonadiabatic transitions between polaritonic and dark states^58^ affect the transport regimes of polaritonic states as a function of their excitonic content, we consider 1024 molecules a good trade-off.

The dispersion of the one-dimensional bidirectional BSW was fitted to the experimental dispersion^17^ with a linear function and discretized into *n*_modes_ = 240 modes^35,39^ (120 modes with positive *k*_*z*_-vectors and 120 modes with negative *k*_*z*_-vectors). The dispersion was tuned to be resonant with the excitation energy of the MeB molecules (dashed line in Figure 2b) at wave vector *k*_*z*_ = 11.41 μm^–1^. With an electric field strength of 0.207 MV cm^–1^, the Rabi splitting between the upper (UP) and lower polariton (LP) branches is $\hbar \Omega_{R}=2\hbar \;g\sqrt{N}=407$ meV, where $g\sqrt{N}$ is the collective coupling strength with *g* the average single-molecule interaction strength that is proportional to the transition dipole moment. With such a vacuum field, the ratio between the standard deviation of the MeB absorption spectrum obtained from the QM/MM MD simulations (σ = 63 meV) and the collective coupling strength is 0.31 at resonance. According to Xiong and co-workers this implies that the LP states range from (*i*) fully delocalized at low *k*_*z*_, via (*ii*) partially delocalized around the wave vectors where BSW and MeB are resonant, to (*iii*) localized at high *k*_*z*_ where the polaritons become similar to the dark states.^59^ In this regime, we, therefore, can anticipate a non-negligible effect of static molecular disorder on polariton transport.^26^ Assuming a rather typical value of σ = 22 meV for J-aggregates,^60,61^ we infer that the ratio in our simulation should be close, if not identical, to that in the experimental study in which a Rabi splitting of ℏΩ_R_ = 142 meV was observed for the strongly coupled J-aggregates.^17^ Because in our simulations we only consider a single layer of molecules, we neglect the *x*-dependence in eq 1. Furthermore, as we restrict ourselves to model one-dimensional transport along a chain of molecules in both the positive and negative *z*-direction, the field distribution becomes *E*(*z*) = *E*_0_e^i*k*_*z*_ *z*^.

In Figure 2c, we show the time evolution of the probability density of the total polaritonic wave function, |Ψ­(*z*,*t*)|^2^, after the excitation of a single MeB molecule, located at 125 μm on the DBR surface. The wave packet rapidly expands, with the fronts of the wave packet moving at the group velocity of the lower polariton, υ_LP_^max^ = 172 μm ps^–1^ (dashed lines in Figure 2c and Figure S2 in Supporting Information, Supporting Information), while most of the packet remains close to the position where the molecule was excited and expands slower. Such spreading of the wave packet suggests that the overall transport mechanism is a combination of ballistic motion and diffusion. To disentangle these two processes, we analyze how polaritonic states contribute to transport as a function of their photonic content. To perform this analysis, we monitor the propagation of adiabatic states within finite windows of energy and *k*_*z*_-vectors, two of which are illustrated as rectangles in Figure 2b.

In Figure 3a,b, we show the propagation of the *partial* wave functions, |Ψ_part_|^2^, associated with states in two windows: *w*_*a*_ from *k*_*z*_ = 9.5 μm^–1^ to 10.0 μm^–1^, and *w*_*b*_ from *k*_*z*_ = 10.75 μm^–1^ to *k*_*z*_ = 11.25 μm^–1^ (purple and orange rectangles in Figure 2b, respectively). The mean squared displacements (MSD) of these partial wave packets are plotted in Figure 3c. The MSD plots for the other windows are shown in Supporting Information (Section 3.1). By fitting the mean squared displacement to the general expression for MSD,

2 $$\mathrm{MSD}\left(t\right)=2D_{\beta }t^{\beta }$$

we determine whether the propagation is ballistic or diffusive from the value of the transport exponent, β. Purely ballistic transport corresponds to β = 2, whereas pure diffusion corresponds to the transport exponent equal to unity with *D*_β_ becoming the diffusion coefficient.

**Figure 3:**
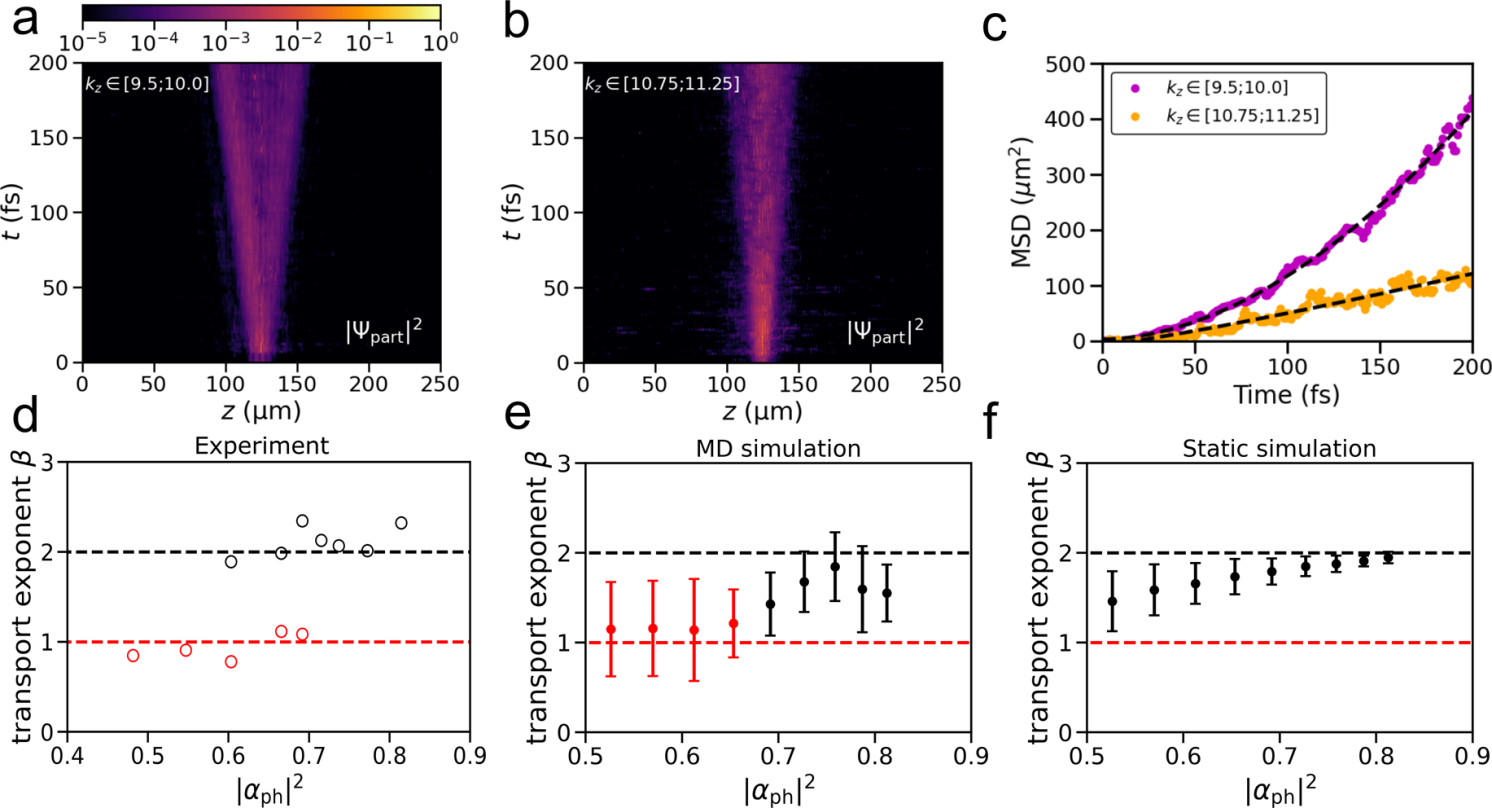
Panels a and b: Probability density, |Ψ_part_|^2^, of the partial wave function extracted from the two windows, which are depicted as purple (a) and orange (b) rectangles in Figure 2b. Panel c: MSD of the partial wave functions from panels a and b. The dashed lines correspond to the fit to MSD = 2*D*_β_ *t* ^β^ with β being the transport exponent. Panels d and e: The transport exponent as a function of the cavity modes contribution |α_ph_|^2^ to polaritonic states, extracted from the experiment (d) and MD simulations (e). Panel d is reproduced from Balasubrahmaniyam et al.^17^ with data provided by Tal Schwartz. Panel f: Values extracted from simulations of two-level systems with static excitation energy disorder of σ = 63 meV. In panels e and f, the transport exponents were extracted from windows of width *Δk*_*z*_ = 0.5 μm^–1^ centered between *k*_*z*_^*c*^ = 9.25 μm^–1^ and *k*_*z*_^*c*^ = 11.25 μm^–1^ with a step of 0.25 μm^–1^ (Table S2). The error bars in panels e and f depict the standard deviations of, respectively, seven and five hundred individual simulations.

In Figure 3e we plot the value of the transport exponent for the partial wave functions extracted from all windows between *k*_*z*_^*c*^ = 9.25 μm^–1^ and *k*_*z*_^*c*^ = 11.25 μm^–1^ as a function of the photonic Hopfield coefficient (|α_ph_|^2^, eq 10) at the center of the windows (Table S2). The plot suggests that shifting the window upward along the lower polariton branch toward states with a lower photonic content is accompanied by a change in the transport exponent, β, from two to one, indicating a transition between ballistic propagation and diffusion, in line with the experiment (Figure 3d).^17^

Note that there are deviations in the transport exponent in the two lowest energy windows. Because the population of the polaritonic states in these windows remains very small due to the large energy gap, we attribute these deviations to numerical noise associated with the normalization of the MSD (Supporting Information, eq 34). Averaging over significantly more than the seven trajectories used in this work could potentially reduce this statistical error but would require more computational resources than currently available. In addition, for each window we also extract the ballistic expansion velocity^17^ and diffusion coefficients and plot these quantities as a function of the photonic content in Figure S5.

The transition from ballistic to diffusive transport cannot be fully captured in simulations of two-level systems with static excitation energy disorder. In these simulations (Section 3.2 in the Supporting Information), the disorder was modeled by randomly drawing the excitation energies of the two-level systems from a Gaussian distribution

3 $$p\left(E\right)=\frac{1}{\sqrt{2\pi }\sigma }\exp\left[-\frac{{\left(E-E_{0}\right)}^{2}}{2\sigma^{2}}\right]$$

where *E*_0_ is the mean value and σ is the “disorder strength” that determines the absorption line width of the disordered two-level system. As shown in Figure 3f, the transport exponent decreases while moving up along the LP branch (decreasing |α_ph_|^2^) in simulations with σ = 63 meV, which matches the line width of the MeB QM/MM model used in our atomistic MD simulations, yet the transition to diffusion is incomplete as β ≈ 1.5 in the lowest energy window.

As reported in early studies on disordered polaritons,^39,62,63^ disorder in the excitation energies can cause coherent backscattering of polaritonic states that disrupts their directional ballistic propagation according to the dispersion. Indeed, while in simulations of static two-level systems with a small disorder (σ = 22 meV) we observe almost no deviation from ballistic transport, a large disorder (σ = 100 meV) leads to a complete transition into a diffusion regime around |α_ph_|^2^ ≈ 0.7 (Figure S9). In the latter simulations, the ratio between the disorder and the collective coupling strength is large ( $\sigma /g\sqrt{N}=0.49$ ), which means that due to the disorder, the system is in a regime where the polaritonic states with lower photonic contents are partially localized on the molecules.^59^ We speculate that in addition to the backscattering process, such localization of the excitation also contributes to making the transport diffusive.

The absence of a transition into the diffusion regime on a 200 fs time scale in simulations of disordered two-level systems on a DBR surface is in line with previous simulations of such systems in Farby–Pérot cavities.^26,29^ To explore whether the transition can occur at a longer time scale, we extended the simulations of the static two-level system with a disorder strength of σ = 63 meV, to *t* = 1 ps. While on this time scale, the transport exponent, extracted from the MSD, increases with |α_ph_|^2^, the values of the transport exponent, β, become progressively smaller with increasing simulation time and can even drop below 1.0 for LP states with very low photonic content (Figure S11). We note that a similar subdiffusive behavior was previously reported in simulations of static two-level molecules with moderate and high disorder.^25,26^

Although the static simulations can yield the transition to diffusion and even subdiffusion, this transition occurs either (*i*) at larger disorder strengths, at which polaritons become partially localized^59^ and presumably enter a transport regime different from that considered here or in the experiment,^17^ or (*ii*) beyond the subpicosecond time scale, at which diffusive propagation of more excitonic-like lower polaritons was observed in both our MD trajectories and microscopy measurements.

The difference between the transport mechanisms observed in the MD and static simulations (Figure 3e,f) suggests that molecular vibrations play an important role in modifying the mechanism of polariton transport along the lower polariton branch on sub-ps time scales. Through displacements along the nonadiabatic coupling vector, these vibrations drive population transfer between polaritonic and dark states.^58^ Although some controversy remains as to whether such vibration-mediated population transfer should be considered a manifestation of radiative pumping (RP),^64,65^ vibrational assisted scattering (VAS),^54,66–68^ or a combination of both,^69^ the strength of the nonadiabatic coupling, and hence also the magnitude of the transfer rate, is inversely proportional to the energy gap between these states.^58,70–72^ Therefore, population exchange between the stationary dark states, which are distributed around the molecular absorption maximum, on the one hand, and the low-energy, highly photonic LP states, on the other hand, is slow, resulting in a long-term ballistic propagation of population in these states. Moving up in energy along the LP branch toward higher *k*_*z*_-vectors reduces the energy gap. Therefore, the transfer rate and, hence, the likelihood of population getting transiently trapped in the dark states manifold increase significantly. Eventually, as the energy gap narrows further, and the polaritonic states start overlapping with the dark states in the exciton reservoir,^59,73–75^ the population exchange between the propagating bright and stationary dark states becomes continuous and reversible, rendering the propagation less ballistic and more diffusive.

To further elucidate the role of molecular vibrations in the observed crossover between different regimes of polariton transport, we repeated the MD simulations of *N* = 1024 MeB molecules in a vacuum, with constraints imposed on all bond lengths^76^ and on the out-of-plane motions of the heavy atoms (Section 3.3 in the Supporting Information). By restricting the displacements along vibrational modes, these constraints reduce the absorption line width and the Stokes shift of the molecules, resulting in a smaller overlap between the dark states and the LP branch. In addition, because the nonadiabatic coupling between the bright polaritonic states (ψ_*m*_) and dark states (ψ_*l*_) depends on the velocity of displacements that overlap with the nonadiabatic coupling vector (i.e., *V*_*m,l*_ = **d**_*m,l*_·**Ṙ**, with $d_{m,l}=\frac{\left\langle \psi_{l}\right|\nabla_{R}{Ĥ}^{\mathrm{TC}}\left|\psi_{m}\right\rangle }{E_{m}-E_{l}}$ and **Ṙ** the velocity),^58^ the constraints also reduce the magnitude of the nonadiabatic coupling, *V*_*m*,*l*_, and, therefore, the rate at which population transfers. As a consequence, population transfer into the dark states is suppressed, the transport becomes ballistic over the full range of the LP branch, and no crossover into a diffusion regime is observed (Figures S13 and S14).

To compensate for a reduction of the overlap between the dark states and the bright polariton states due to narrowing of the absorption line width from σ = 63 meV to σ = 28 meV, we also repeated MD simulations with constraints on molecular motions at a smaller Rabi splitting of ℏΩ_R_ = 181 meV. This value of the Rabi splitting provides the same ratio between the standard deviation of the molecular absorption spectrum and the collective coupling strength, $\sigma /g\sqrt{N}=0.31$ , as in the experiment^17^ and in the MD simulations without constraints and ℏΩ_R_ = 407 meV. The results of these simulations suggest ballistic propagation of lower polaritons in all energy and wave-vector windows (Figure S17), further emphasizing the importance of molecular vibrations for observing the crossover from ballistic transport to diffusion.

In addition, we also performed simulations of two-level systems with quasi-dynamic disorder, in which excitation energies were drawn from the Gaussian distribution (eq 3) and resampled every several femtoseconds in order to imitate the continuous energy redistribution due to dynamics of molecules (Section S3.4 in the Supporting Information). The resampling intervals τ were drawn from a Poisson distribution:^77^

4 $$p\left(\tau \right)=\frac{\langle \tau \rangle^{\tau }e^{-\left\langle \tau \right\rangle }}{\tau !}$$

where ⟨τ⟩ is the average resampling time. As in simulations with static disorder, an incomplete transition to diffusion is observed in these quasi-dynamic simulations, and the transport exponent remains closer to two for most of the *k*_*z*_-vectors windows (Figure S17). This finding indicates that the transition on a short time scale of several hundred femtoseconds cannot be caused by the excitation energy disorder alone and hence further emphasizes the role of molecular vibrations in this process.

To conclude, we performed molecular dynamics simulations of polariton transport in a system of methylene blue dye molecules strongly coupled to the Bloch surface wave on a distributed Bragg reflector. In line with experiment,^17^ our simulations reveal a transition between ballistic and diffusive propagation as the photonic contribution to the polaritonic states decreases. Importantly, an incomplete transition was observed in simulations in which the molecules were frozen and modeled as two-level systems. The lack of a transition in such model systems underscores the role of molecular vibrations in changing the transport regime along the LP branch.

## Simulation Details

Although polaritons and their optical characteristics emerge as the solutions of the classical Maxwell’s equations,^66^ such macroscopic approach does not break the translational symmetry due to structural disorder in the coupled material, which is modeled as a phenomenological broadening of the material’s excitation energy. We therefore used a microscopic model instead,^18,39^ in which the material degrees of freedom are modeled explicitly at the hybrid quantum mechanics/molecular mechanics (QM/MM) level of theory^41^ and the dynamics of the combined nuclear, electronic and BSW mode degrees of freedom are propagated by means of semiclassical molecular dynamics (MD).

In our MD simulations, we apply the Born–Oppenheimer approximation^34,78^ to separate the nuclear degrees of freedom, which are treated classically, from the electronic plus photonic degrees of freedom, which are treated quantum mechanically with the QM/MM extension^35^ of the Tavis–Cummings Hamiltonian:^79,80^

5 $${Ĥ}^{\mathrm{TC}}=\overset{N}{\underset{j}{\sum }}\hbar \omega_{\mathrm{exc}}\left(R_{j}\right){\mathrm{\sigma ̂}}_{j}^{+}{\mathrm{\sigma ̂}}_{j}^{-}+\overset{N}{\underset{j}{\sum }}V_{S_{0}}\left(R_{j}\right)+\overset{n_{\mathrm{modes}}}{\underset{p}{\sum }}\hbar \omega_{\mathrm{cav}}\left(k_{z,p}\right){â}_{p}^{†}{â}_{p}-\overset{N}{\underset{j}{\sum }}\overset{n_{\mathrm{modes}}}{\underset{p}{\sum }}\sqrt{\frac{\hbar \omega_{\mathrm{cav}}\left(k_{z,p}\right)}{2\epsilon_{0}V}}\mu \left(R_{j}\right)\cdot \left[e^{ik_{z,p}z_{j}}{\mathrm{\sigma ̂}}_{j}^{+}{â}_{p}+e^{-ik_{z,p}z_{j}}{\mathrm{\sigma ̂}}_{j}^{-}{â}_{p}^{†}\right]$$

where σ̂_*j*_^+^ = |S_1_^*j*^⟩⟨S_0_^*j*^| is an operator that excites molecule *j* with nuclear coordinates **R**_*j*_ from the electronic ground state |S_0_^*j*^⟩ with energy *V*_S_0__(**R**_*j*_) into the first electronic excited state |S_1_^*j*^⟩ with energy *V*_S_1__(**R**_*j*_) . Accordingly, the excitation energy is defined as ℏω_exc_(**R**_*j*_) = *V*_S_1__(**R**_*j*_) – *V*_S_0__(**R**_*j*_) . Likewise, σ̂_*j*_^–^ = |S_0_^*j*^⟩⟨S_1_^*j*^| de-excites molecule *j* from electronic excited state |S_1_^*j*^⟩ into the electronic ground state |S_0_^*j*^⟩. Operators *â*_*p*_^†^ and *â*_*p*_ create and annihilate a photon of energy ℏω_cav_(**k**_*z*,*p*_) in BSW mode *p* with in-plane momentum **k**_*z*,*p*_ along the surface of the DBR. Finally, **μ**(**R**_*j*_) is the transition dipole moment of molecule *j*, and *z*_*j*_ is the position of molecule *j* on the surface of the DBR structure.

The Ehrenfest molecular dynamics approach is used to model the classical degrees of freedom,^81^ which evolve on a potential energy surface that is the expectation value of the energy of the total wave function of the quantum degrees of freedom: *V*(**R**) = ⟨Ψ|*Ĥ*|Ψ⟩. The total wave function, |Ψ­(*t*)⟩, is propagated along the classical trajectory as a linear combination of diabatic product states between the *N* molecular excitations and *n*_modes_ BSW modes:^37^

6 $$\left|\Psi \left(t\right)\right\rangle =\overset{N+n_{\mathrm{modes}}}{\underset{j}{\sum }}d_{j}\left(t\right)\left|\phi_{j}\right\rangle$$

with

7 $$\left|\phi_{j}\right\rangle ={\mathrm{\sigma ̂}}_{j}^{+}\left|S_{0}^{1}S_{0}^{2}...S_{0}^{N-1}S_{0}^{N}\right\rangle ⊗\left|00..0\right\rangle$$

for 1 ≤ *j* ≤ *N*, and

8 $$\left|\phi_{j>N}\right\rangle ={â}_{j-N}^{†}\left|S_{0}^{1}S_{0}^{2}...S_{0}^{N-1}S_{0}^{N}\right\rangle ⊗\left|00..0\right\rangle$$

for *N* < *j* ≤ *N* + *n*_modes_. State |ϕ_*j*_⟩ corresponds to molecule *j* in its first electronic excited state (S_1_^*j*^), while the other molecules are in the ground state (S_0_^*i*≠*j*^) and the photonic states are empty, whereas state |ϕ_*j*>*N*_⟩ corresponds to one of the BSW modes excited, with all molecules in the electronic ground state. In eq 6, *d*_*j*_(*t*) are the time-dependent expansion coefficients of the total wave function, which reflect the population of each molecular excitation and each photonic mode during the evolution of the system. These coefficients are propagated with a unitary propagator.^82^ Further details of the simulation method can be found in the Supporting Information (Section 1) or in previous publications.^34,35,37^

In the experiment of Balasubrahmaniyam et al., the molecules were optically excited at a wavelength below the excitation maximum and hence into a local molecular excited state that is energetically above the bright S_1_ state that was coupled to the BSW. While within the focal spot, the pump pulse can excite multiple molecules simultaneously, the relaxation of these molecules into the local S_1_ minimum (i.e., Kasha’s rule^83^) is assumed to be an incoherent process, in particular at room temperature, at which the molecular environment is significantly disordered. To mimic such initial conditions, we prepare the MeB-BSW system in the first excited electronic state (S_1_) of a single MeB molecule, located at the center of a 250 μm periodic DBR surface (Figure 2a), and run multiple (seven) simulations with different starting velocities.

The Ehrenfest QM/MM trajectories are computed for 200 fs with an integration time step of 0.5 fs. The temperature is kept constant at 300 K with the v-rescale thermostat.^84^ Because these MD trajectories are run for much shorter than the typical lifetime of a BSW, we neglect decay in our simulations. Further details of the simulations performed in this article are presented in the Supporting Information.

For the analysis of the trajectories, we also expand the total time-dependent wave function in the basis of the eigenstates of the Tavis–Cummings Hamiltonian, as follows:

9 $$\left|\Psi \left(t\right)\right\rangle =\underset{j}{\sum }c_{m}\left(t\right)\left|\psi_{m}\right\rangle$$

where

10 $$\left|\psi_{m}\right\rangle =\left(\overset{N}{\underset{j}{\sum }}\beta_{j}^{m}{\mathrm{\sigma ̂}}_{j}^{+}+\overset{n_{\mathrm{modes}}}{\underset{p}{\sum }}\alpha_{p}^{m}{â}_{p}^{†}\right)\left|S_{0}^{1}S_{0}^{2}...S_{0}^{N-1}S_{0}^{N}\right\rangle ⊗\left|0\right\rangle$$

Here, |ψ_*m*_⟩ are time-independent eigenstates of the Tavis–Cummings Hamiltonian (eq 5), which parametrically depend on the coordinates of the nuclei, and hence are adiabatic. The β_*j*_^*m*^ and α_*p*_^*m*^ expansion coefficients denote contributions of the molecular excitons (|S_1_^*j*^⟩) and of the photonic modes (|1_*p*_⟩) to the adiabatic eigenstates |ψ_*m*_⟩. These coefficients are obtained at each time step along with the eigenstates |ψ_*m*_⟩ by diagonalizing the matrix representation of *Ĥ* ^TC^ (eq 5) in the basis of the diabatic product states (eqs 7 and 8) while keeping the nuclear coordinates fixed.

The time-dependent expansion coefficients, *c*_*m*_(*t*), in this adiabatic representation are thus related to the time-dependent expansion coefficients, *d*_*j*_(*t*), in the diabatic representation (eq 6) via the unitary matrix, **U**, that diagonalizes the Tavis–Cummings matrix (i.e., *c*_*m*_(*t*) = ∑_*j*_^*N+n*_modes_^ *U*_*mj*_^†^ *d*_*j*_(*t*), with *U*_*jm*_ = β_*j*_^*m*^ if *j* ≤ *N* and *U*_*jm*_ = α_*j*–*N*_^*m*^ if *j* > *N*).

In a perfectly ordered system, there are eigenstates either with or without cavity mode contributions called the polaritonic bright and dark states, respectively. However, in a disordered system, the cavity modes contribute to all eigenstates.^74,85^ To make a distinction between bright and dark states also in such situations, we apply a numerical threshold^39^ and consider a state bright if the total contribution of the cavity modes exceeds 2% and dark otherwise.^73^

To explore how the photonic character of the adiabatic eigenstates affects their contribution to the overall transport, we decompose the total wave function into *partial* wave functions, |Ψ_*w*_^part^(*t*)⟩, within a narrow range of wave vectors. These partial wave functions are linear combinations of the eigenstates (eq 10) in fixed intervals of wave vectors and energies. Each interval, called a window, *w*_*i*_, is centered at *k*_*z,i*_^*c*^ and *E*_*c*_ and ranges from *k*_*z,i*_^min^ to *k*_*z,i*_^max^ and from *E*_min_ to *E*_max_ (Table S2). The expansion coefficients, *c*_*m*∈*w*_*i*__(*t*), of the partial wave functions are thus obtained by projecting the adiabatic states within a window onto the total time-dependent wave function in the adiabatic representation (eq 9).

## Supplementary Material



## Notes

Details on the multiscale molecular dynamics model, simulation details, analysis of the wave packets and results of additional simulations, supporting figures and tables (PDF)
