# Supplementary material for: Disentangling Enhanced Diffusion and Ballistic Motion of Excitons Coupled to Bloch Surface Waves with Molecular Dynamics Simulations
Source: J Phys Chem Lett. 2025 Jun 24;16(26):6719–27. doi: 10.1021/acs.jpclett.5c01391 (PMC12604612; doi:10.1021/acs.jpclett.5c01391)
Supplement: Supplementary file 1 [file jz5c01391_si_001.pdf]

Supporting information

for

Disentangling enhanced diffusion and ballistic motion of excitons coupled to Bloch surface waves with molecular dynamics simulations

Ilia Sokolovskii,* Yunyi Luo, and Gerrit Groenhof

Nanoscience Center and Department of Chemistry, University of Jyväskylä, P.O. Box 35, 40014 Jyväskylä, Finland.

E-mail: ilia.sokolovskii@jyu.fi

Contents

1 Molecular Dynamics Simulation Model **3**

1.1 Multiscale Tavis-Cummings Hamiltonian . . . 3

1.2 One-dimensional photonic crystal . . . 4

1.3 Ehrenfest dynamics . . . 8

1.4 Resolving polariton transport in energy/momentum space . . . 9

2 Simulation details **13**

2.1 Methylene Blue model . . . 13

2.2 MeB-BSW system . . . 15

3 Analysis of the wave packets **18**

3.1 Simulation of $N = 1024$ molecules . . . 18

3.1.1 Partial wave packet . . . 18

3.1.2 Estimation of transport velocities and diffusion coefficients . . . 21

3.2 Simulation of two-level systems with static disorder . . . 22

3.3 MD simulations with constraints on bond lengths and out-of-plane motions of Methylene Blue molecules . . . 30

3.3.1 $\hbar\Omega_{\mathrm{R}} = 407$ meV . . . 30

3.3.2 $\hbar\Omega_{\mathrm{R}} = 181$ meV . . . 34

3.4 Simulation of two-level systems with quasi-dynamic disorder . . . 38

References **42**

1 Molecular Dynamics Simulation Model

1.1 Multiscale Tavis-Cummings Hamiltonian

In our model, strong coupling between *electronic* excitations of molecules and light modes of a cavity is described within the Born-Oppenheimer approximation, in which fast electronic *plus* photonic degrees of freedom are separated from slow nuclear degrees of freedom[1]. To describe the electronic-photonic degrees of freedom, we extend the traditional Tavis-Cummings Hamiltonian[2,3] to the case of multiple cavity modes, n_{modes}, and replace two-level systems with adiabatic electronic states of the molecules[4]:

$$\begin{aligned}\hat{H}^{\text{TC}} \quad = \quad & \textstyle\sum_j^N \hbar\omega_{\text{exc}}(\mathbf{R}_j)\hat{\sigma}_j^+\hat{\sigma}_j^- + \sum_{k_z}^{n_{\text{modes}}} \hbar\omega_{\text{cav}}(k_z)\hat{a}_{k_z}^\dagger \hat{a}_{k_z} + \\ & \textstyle\sum_j^N \sum_{k_z}^{n_{\text{modes}}} \hbar g_j(k_z)\left(\hat{\sigma}_j^+\hat{a}_{k_z}e^{ik_z z_j} + \hat{\sigma}_j^-\hat{a}_{k_z}^\dagger e^{-ik_z z_j}\right) + \\ & \textstyle\sum_i^N V_{\text{S}_0}^{\text{mol}}(\mathbf{R}_i).\end{aligned} \tag{1}$$

In Equation 1, the operator $\hat{\sigma}_j^+$ ($\hat{\sigma}_j^-$) excites (de-excites) molecule j from the electronic ground (excited) state $|\text{S}_0^j(\mathbf{R}_j)\rangle$ ($|\text{S}_1^j(\mathbf{R}_j)\rangle$) to the electronic excited (ground) state $|\text{S}_1^j(\mathbf{R}_j)\rangle$ ($|\text{S}_0^j(\mathbf{R}_j)\rangle$); $\mathbf{R}_j$ is the vector of the Cartesian coordinates of all atoms in molecule j, centered at z_j; the operators $\hat{a}_{k_z}^\dagger$ and $\hat{a}_{k_z}$, respectively, create and annihilate a cavity mode excitation with wave-vector k_z; $\hbar\omega_{\text{exc}}(\mathbf{R}_j)$ is the excitation energy of molecule j, defined as:

$$\hbar\omega_{\text{exc}}(\mathbf{R}_j) = V_{\text{S}_1}^{\text{mol}}(\mathbf{R}_j) - V_{\text{S}_0}^{\text{mol}}(\mathbf{R}_j), \tag{2}$$

where $V_{\text{S}_0}^{\text{mol}}(\mathbf{R}_j)$ and $V_{\text{S}_1}^{\text{mol}}(\mathbf{R}_j)$ are the adiabatic potential energy surfaces (PESs) of molecule j in the electronic ground (S_0) and excited (S_1) states, respectively. The last term in Equation 1 is the total potential energy of the system in the absolute ground state (*i.e.*, with all molecules and cavity modes de-excited), defined as the sum of the ground-state potential energies of all molecules in the

cavity. The $V_{S_0}^{mol}(\mathbf{R}_j)$ and $V_{S_1}^{mol}(\mathbf{R}_j)$ adiabatic PESs are modelled at the hybrid quantum mechanics / molecular mechanics (QM/MM) level of theory[5,6].

The third term in Equation 1 describes the light-matter interaction within the long-wavelength and rotating wave approximations:

$$g_j(k_z) = -\boldsymbol{\mu}(\mathbf{R}_j) \cdot \mathbf{u}_{\text{cav}} \sqrt{\frac{\hbar\omega_{\text{cav}}(k_z)}{2\epsilon_0 V_{\text{cav}}}} \tag{3}$$

with $\boldsymbol{\mu}(\mathbf{R}_j)$ the transition dipole moment of molecule j that depends on the molecular geometry $(\mathbf{R}_j)$; $\mathbf{u}_{\text{cav}}$ the unit vector in the direction of the electric component of cavity vacuum field (*i.e.*, $|\mathbf{E}| = \sqrt{\hbar\omega_{\text{cav}}(k_z)/2\epsilon_0 V_{\text{cav}}}$); ϵ_0 the vacuum permittivity; and V_{cav} the cavity mode volume.

1.2 One-dimensional photonic crystal

The distribution of the electric field in a one-dimensional distributed Bragg reflector (DBR), or photonic crystal (Figure 1 in the main text), with periodic permittivity, $\varepsilon(x) = \varepsilon(x + A)$ with period A, can be found by solving the wave equation[7]

$$\frac{c^2}{\varepsilon(x)} \frac{\partial^2 E(x,t)}{\partial x^2} = -\frac{\partial^2 E(x,t)}{\partial t^2}, \tag{4}$$

where c is the speed of light. Representing the electric field as a product of coordinate-dependent and time-dependent functions, $E(x,t) = E(x)e^{-i\omega t}$, leads to the following eigenvalue equation:

$$\frac{1}{\varepsilon(x)} \frac{\partial^2 E(x)}{\partial x^2} = \frac{\omega^2}{c^2} E(x). \tag{5}$$

According to Bloch-Floquet theorem, a solution of Equation 5 can be chosen as

$$E_K(x) = u_K(x) e^{iKx}, \tag{6}$$

where $u_k(x) = u_k(x+A)$ is a periodic envelope function and K is the so-called Bloch wave number. For a Bloch surface wave, the Bloch wave number is complex and can be written as $K = i|K|$. Therefore, Equation 6 transforms into

$$E_K(x) = u_K(x)e^{-|K|x}. \quad (7)$$

Thus, the electric field strength of the BSW is a decaying periodic function in the photonic crystal (black line for $x < 0$ in Figure 1 in the main text). In the air, the electric field is not modulated by the periodicity of the dielectric structure and decays as (black line for $x > 0$ in Figure 1 in the main text)

$$E_K(x) = E_0 e^{-|K|x} \quad (8)$$

with E_0 the electric field strength at the surface of the photonic crystal.

While the electric field of the BSW is evanescent in the x-direction, there is no restriction on the field distribution along the surface of the DBR, *i.e.* in the y- and z-directions. Therefore, light can freely propagate along the surface as a plane wave, and the electric field can be written as

$$E(x, y, z) = E_0 e^{-|K|x} e^{i(k_y y + k_z z)}. \quad (9)$$

In the current work, we only consider a single layer of molecules and hence disregard the x-dependence in equation 9. Furthermore, we restrict ourselves to model one-dimensional transport along a chain of molecules in the z-direction. With these simplifications, the field distribution becomes

$$E(z) = E_0 e^{ik_z z}. \quad (10)$$

Following Michetti and La Rocca,[8] we impose periodic boundary conditions in the z-direction of the DBR and thus restrict the wave vectors, k_z, to discrete values: $k_{z,p} = 2\pi p/L_z$ with $p \in \mathbb{Z}$ and

L_z the width of the DBR slab. With this approximation, the molecular Tavis-Cummings Hamiltonian in Equation 1 can be represented as an $(N + n_{\text{modes}})$ by $(N + n_{\text{modes}})$ matrix with four blocks[4]:

$$\mathbf{H}^{\text{TC}} = \begin{pmatrix} \mathbf{H}^{\text{mol}} & \mathbf{H}^{\text{int}} \\ \mathbf{H}^{\text{int}\dagger} & \mathbf{H}^{\text{cav}} \end{pmatrix}. \tag{11}$$

We compute the elements of this matrix in the product basis of adiabatic molecular states times cavity mode excitations:

$$\begin{aligned} |\phi_j\rangle &= \hat{\sigma}_j^+|\text{S}_0^1\text{S}_0^2..\text{S}_0^{N-1}\text{S}_0^N\rangle \otimes |00..0\rangle \\ &= \hat{\sigma}_j^+|\Pi_i^N \text{S}_0^i\rangle \otimes |\Pi_k^{n_{\text{modes}}} 0_k\rangle \\ &= \hat{\sigma}_j^+|\phi_0\rangle \end{aligned} \tag{12}$$

for $1 \leq j \leq N$, and

$$\begin{aligned} |\phi_{j>N}\rangle &= \hat{a}_{j-N}^\dagger|\text{S}_0^1\text{S}_0^2..\text{S}_0^{N-1}\text{S}_0^N\rangle \otimes |00..0\rangle \\ &= \hat{a}_{j-N}^\dagger|\Pi_i^N \text{S}_0^i\rangle \otimes |\Pi_k^{n_{\text{modes}}} 0_k\rangle \\ &= \hat{a}_{j-N}^\dagger|\phi_0\rangle \end{aligned} \tag{13}$$

for $N < j \leq N + n_{\text{modes}}$. In these expressions, $|00..0\rangle$ indicates that the Fock states for all n_{modes} cavity modes are empty. The basis state $|\phi_0\rangle$ is the ground state of the molecule-cavity system with no excitations of neither the molecules nor cavity modes:

$$|\phi_0\rangle = |\text{S}_0^1\text{S}_0^2..\text{S}_0^{N-1}\text{S}_0^N\rangle \otimes |00..0\rangle = |\Pi_i^N \text{S}_0^i\rangle \otimes |\Pi_k^{n_{\text{modes}}} 0_k\rangle. \tag{14}$$

The upper left block, $\mathbf{H}^{\text{mol}}$, is an $N \times N$ matrix containing the single-photon excitations of the molecules. Because we neglect direct excitonic interactions between molecules, this block is diagonal, with elements labelled by the molecule indices j:

$$H^{\text{mol}}_{j,j} = \langle \phi_0 | \hat{\sigma}_j \hat{H}^{\text{TC}} \hat{\sigma}^+_j | \phi_0 \rangle \tag{15}$$

for $1 \leq j \leq N$. Each matrix element of $\mathbf{H}^{\text{mol}}$ thus represents the potential energy of a molecule, j, in the electronic excited state $|\text{S}^j_1(\mathbf{R}_j)\rangle$, while all other molecules, $i \neq j$, are in the electronic ground state $|\text{S}^i_0(\mathbf{R}_i)\rangle$:

$$H^{\text{mol}}_{j,j} = V^{\text{mol}}_{\text{S}_1}(\mathbf{R}_j) + \sum_{i \neq j}^{N} V^{\text{mol}}_{\text{S}_0}(\mathbf{R}_i). \tag{16}$$

The lower right block, $\mathbf{H}^{\text{cav}}$, is an $n_{\text{modes}} \times n_{\text{modes}}$ matrix (with $n_{\text{modes}} = n_{\text{max}} - n_{\text{min}} + 1$) containing the single-photon excitations of the cavity (BSW) modes, and is also diagonal:

$$H^{\text{cav}}_{p,p} = \langle \phi_0 | \hat{a}_p \hat{H}^{\text{TC}} \hat{a}^\dagger_p | \phi_0 \rangle \tag{17}$$

for $n_{\text{min}} \leq p \leq n_{\text{max}}$. Here, $\hat{a}^\dagger_p$ excites cavity mode p with wave-vector $k_{z,p} = 2\pi p / L_z$. In these matrix elements, all molecules are in the electronic ground state $|\text{S}^i_0(\mathbf{R}_i)\rangle$. The energy is therefore the sum of the cavity energy at $k_{z,p}$ and the molecular ground state energies:

$$H^{\text{cav}}_{p,p} = \hbar\omega_{\text{cav}}(k_{z,p}) + \sum_{j}^{N} V^{\text{mol}}_{\text{S}_0}(\mathbf{R}_j), \tag{18}$$

where $\omega_{\text{cav}}(k_{z,p})$ is the cavity dispersion (dashed-dotted curve in Figure 2**b**, main text). In this work, the dispersion of the BSW was fitted to reproduce the experimental dispersion from the study of Balasubrahmaniyam *et. al.*[9].

The two $N \times n_{\text{modes}}$ off-diagonal blocks $\mathbf{H}^{\text{int}}$ and $\mathbf{H}^{\text{int}\dagger}$ in the multi-mode Tavis-Cummings Hamiltonian (Equation 11) model the light-matter interactions between the molecules and the cavity modes. Within the long-wavelength approximation these matrix elements can be approximated by

the overlap between the transition dipole moment of molecule j and the transverse electric field of cavity mode p at the geometric center z_j of that molecule:

$$
\begin{aligned}
H^{\text{int}}_{j,p} &= -\boldsymbol{\mu}(\mathbf{R}_j)\cdot\mathbf{u}_{\text{cav}}\sqrt{\tfrac{\hbar\omega_{\text{cav}}(k_{z,p})}{2\epsilon_0 V_{\text{cav}}}}\langle\phi_0|\hat{\sigma}_j\hat{\sigma}_j^{+}\hat{a}_p e^{i2\pi p z_j/L_z}\hat{a}_p^{\dagger}|\phi_0\rangle \\
&= -\boldsymbol{\mu}(\mathbf{R}_j)\cdot\mathbf{u}_{\text{cav}}\sqrt{\tfrac{\hbar\omega_{\text{cav}}(k_{z,p})}{2\epsilon_0 V_{\text{cav}}}}e^{i2\pi p z_j/L_z}
\end{aligned}
\tag{19}
$$

for $1 \leq j \leq N$ and $n_{\text{min}} \leq p \leq n_{\text{max}}$.

1.3 Ehrenfest dynamics

In our simulations, classical trajectories evolve under the influence of the expectation value of forces with respect to the polaritonic wave function[10], while the polaritonic wave function evolves along with the classical degrees of freedom. By expanding the total wave function as a linear combination of the *time-independent* diabatic light-matter states (Equations 12 and 13),

$$
|\Psi(t)\rangle = \sum_{j}^{N+n_{\text{modes}}} d_j(t)|\phi_j\rangle, \tag{20}
$$

the evolution of the *time-dependent* diabatic expansion coefficients, $d_j(t)$, is obtained by numerically integrating the Schrödinger equation over discrete time intervals, Δt,

$$
\mathbf{d}(t+\Delta t) = \mathbf{P}^{\text{dia}}\mathbf{d}(t). \tag{21}
$$

Here, $\mathbf{d}(t)$ is a vector containing the diabatic expansion coefficients $d_j(t)$ and $\mathbf{P}^{\text{dia}}$ is the propagator in the diabatic basis[11]:

$$
\mathbf{P}^{\text{dia}} = \exp\left[-i\left(\mathbf{H}^{\text{TC}}(t+\Delta t) + \mathbf{H}^{\text{TC}}(t) - i\hbar\boldsymbol{\gamma}\right)\Delta t/2\hbar\right], \tag{22}
$$

where $\boldsymbol{\gamma}$ is a vector containing decay rates γ_j of each molecule and each cavity mode. Because in the current work, we simulate a short-time propagation of exciton-polaritons during two hundred femtoseconds, which is shorter than the lifetimes of both singlet excitons in Methylene Blue (MeB) molecule used in this study and Bloch surface waves[12–14], we neglect the decay rate vector $\boldsymbol{\gamma}$ in Equation 22, which results in

$$\mathbf{P}^{\text{dia}} = \exp\left[-i\left(\mathbf{H}^{\text{TC}}(t+\Delta t) + \mathbf{H}^{\text{TC}}(t)\right)\Delta t/2\hbar\right]. \tag{23}$$

1.4 Resolving polariton transport in energy/momentum space

To imitate an experimental approach[9], in which the transport of polaritonic states was resolved by probing their propagation at an energy and wave vector that match their locations along the lower BSW-polariton dispersion curve, we diagonalize the Tavis-Cummings Hamiltonian matrix (Equation 11) at each time step of simulation to obtain *adiabatic* polariton wave functions:

$$|\psi^m\rangle = \left(\sum_j^N \beta_j^m \hat{\sigma}_j^+ + \sum_p^{n_{\text{modes}}} \alpha_p^m \hat{a}_p^\dagger\right)|\phi_0\rangle = \sum_i^{N+n_{\text{modes}}} U_{im}|\phi_i\rangle \tag{24}$$

with β_j^m and α_p^m being the expansion coefficients, which reflect, respectively, the contribution of the molecular excitons and the cavity mode excitations to polariton state $|\psi^m\rangle$. Because the polariton states form a complete set, the total wave function in Equation 20 can be equivalently represented as a linear combination of these states:

$$|\Psi(t)\rangle = \sum_m^{N+n_{\text{modes}}} c_m(t)|\psi^m\rangle. \tag{25}$$

Here, the time-dependent expansion coefficients, $c_m(t)$, associated with polariton states, are related to the diabatic expansion coefficients, $d_j(t)$ in Equation 20, via the unitary transformation:

$$\mathbf{c}(t) = \mathbf{U}^{-1}\mathbf{d}(t) = \mathbf{U}^\dagger\mathbf{d}(t) \tag{26}$$

with $\mathbf{U}$ the unitary matrix that diagonalizes the Hamiltonian matrix and contains elements U_{im} (Equation 24).

To visualise polariton propagation, the probability density of the total wave function $|\Psi(t)|^2$ was constructed at each time step as a sum of excitonic $|\Psi_{\text{exc}}|^2$ and photonic $|\Psi_{\text{phot}}|^2$ contributions[15]. The amplitude of $|\Psi_{\text{exc}}(t)\rangle$ at position z_j of molecule j (with $z_j = (j-1)L_z/N$ for $1 \leq j \leq N$) was obtained by projecting the excitonic basis state in which molecule j at position z_j is excited, onto the total wave function (Equation 20):

$$|\Psi_{\text{exc}}(z_j,t)\rangle = (\hat{\sigma}_j^+|\phi_0\rangle\langle\phi_0|\hat{\sigma}_j)|\Psi(t)\rangle = \sum_m^{N+n_{\text{modes}}} c_m(t)\beta_j^m\hat{\sigma}_j^+|\phi_0\rangle. \tag{27}$$

The cavity mode excitations are described as plane waves that are delocalised in real space. We therefore obtained the amplitude of the cavity mode excitations in polaritonic eigenstate $|\psi^m\rangle$ at position z_j by Fourier transforming the projection of the cavity mode Fock states, in which cavity mode p is excited, onto $|\psi^m\rangle$:

$$|\psi_{\text{phot}}^m(z_j)\rangle = \mathcal{FT}^{-1}\left[\sum_p^{n_{\text{modes}}}(\hat{a}_p^\dagger|\phi_0\rangle\langle\phi_0|\hat{a}_p)|\psi^m\rangle\right] = \frac{1}{\sqrt{N}}\sum_p^{n_{\text{modes}}}\alpha_p^m e^{ik_{z,p}z_j}\hat{a}_p^\dagger|\phi_0\rangle. \tag{28}$$

The total contribution of the cavity mode excitations to the wavepacket at position z_j at time t was then obtained as the weighted sum over the Fourier transforms:

$$\begin{aligned}|\Psi_{\text{phot}}(z_j,t)\rangle &= \textstyle\sum_m^{N+n_{\text{modes}}} c_m(t)\times\mathcal{FT}^{-1}\left[\sum_p^{n_{\text{modes}}}(\hat{a}_p^\dagger|\phi_0\rangle\langle\phi_0|\hat{a}_p)|\psi^m\rangle\right]\\ &= \textstyle\sum_m^{N+n_{\text{modes}}} c_m(t)\frac{1}{\sqrt{N}}\sum_p^{n_{\text{modes}}}\alpha_p^m e^{ik_{z,p}z_j}\hat{a}_p^\dagger|\phi_0\rangle.\end{aligned} \tag{29}$$

The propagation of polaritonic states within a k_z-vector/energy window, w, was obtained by constructing *partial* excitonic and photonic wave functions, in which, rather than summing over all

$N + n_{\text{modes}}$ polaritonic states, only lower polariton states with eigenenergies and wave vectors lying in a certain window of size $\Delta E \times \Delta k_z$ were included in the sum:

$$|\Psi^{\text{part}}_{\text{exc},w}(z_j,t)\rangle = \sum_{m' \in w}^{n_{\text{modes}}} c_{m'}(t)\beta_j^{m'}\hat{\sigma}_j^{+}|\phi_0\rangle. \tag{30}$$

and

$$|\Psi^{\text{part}}_{\text{phot},w}(z_j,t)\rangle = \frac{1}{\sqrt{N}}\sum_{m' \in w}^{n_{\text{modes}}} c_{m'}(t)\sum_{p}^{n_{\text{modes}}} \alpha_p^{m'} e^{ik_{z,p}z_j}\hat{a}_p^{\dagger}|\phi_0\rangle, \tag{31}$$

for $\{m'|E_{m'} \in \left[E_c - \Delta E/2; E_c + \Delta E/2\right]\}$ and $\{m'|k_z^{m'} \in \left[k_z^c - \Delta k_z/2; k_z^c + \Delta k_z/2\right]\}$ with E_c and k_z^c, respectively, the energy and wave vector, which correspond to the centre of the window and are taken from the ideal BSW-polariton dispersion without excitation energy disorder. $E_{m'}$ is the eigenenergy of polariton state $|\psi^{m'}\rangle$, and $k_z^{m'}$ is the expectation value of the in-plane momentum of polariton state $|\psi^{m'}\rangle$, evaluated as

$$\langle k_z^{m'}\rangle = \frac{\sum_p^{n_{\text{modes}}} |\alpha_p^{m'}|^2 k_{z,p}}{\sum_p^{n_{\text{modes}}} |\alpha_p^{m'}|^2} \tag{32}$$

with $k_{z,p} = 2\pi p/L_z$. By restricting the sums over m' to the first $n_{\text{modes}} = 240$ eigenstates, the analysis only includes the states of the lower polaritonic branch. Finally, the probability amplitude of the partial wave function in window w was computed as a sum of the probability amplitudes of the partial excitonic and photonic contributions:

$$|\Psi^{\text{part}}_{w}(z,t)|^2 = |\Psi^{\text{part}}_{\text{exc},w}(z,t)|^2 + |\Psi^{\text{part}}_{\text{phot},w}(z,t)|^2. \tag{33}$$

As a measure of polariton transport, we calculated the mean squared displacement (MSD) of the partial wave function,

$$\text{MSD}_w(t) = \frac{\langle\Psi^{\text{part}}_{w}(z,t)|\left(\hat{z}(t)-\hat{z}(0)\right)^2|\Psi^{\text{part}}_{w}(z,t)\rangle}{\langle\Psi^{\text{part}}_{w}(z,t)|\Psi^{\text{part}}_{w}(z,t)\rangle}. \tag{34}$$

To define the regime of polariton transport, the MSD was fitted with the following expression:

$$\mathrm{MSD}_w(t) = D_\beta t^\beta, \tag{35}$$

where D_β is a pre-factor and β is the transport exponent that characterizes the propagation of the lower polaritonic states within k_z-vector/energy window w. Subdiffusion is characterized by $\beta < 1$; diffusion by $\beta = 1$; super-diffusion by $1 < \beta < 2$; ballistic transport by $\beta = 2$; and hyper-ballistic transport by $\beta > 2$.

2 Simulation details

2.1 Methylene Blue model

The Amber03 force field was used to model the interactions between MeB and the water solvent, which was described with the TIP3P water model[16]. Atom types and partial charges for the MeB atoms (Figure S1) are listed in Table S1. The partial charges of the atoms were derived following the procedure recommended for the Amber03 force field[17,18]. First, the geometry of MeB was minimized at the HF/6-31G** level of *ab initio* theory, using the IEFPCM continuum solvent model with a relative dielectric constant of 4.0[19]. After the geometry optimizations, the electrostatic potential at 10 concentric layers of 17 points per unit area around each atom was evaluated using the electron density calculated at the B3LYP/cc-pVTZ level of DFT theory[20], again using the IEFPCM continuum solvent model with a relative dielectric constant of 4.0[19]. The atomic charges were obtained by performing a two-stage RESP fit to the electrostatic potential[18], the first without symmetry constraints, and the second with symmetry constraints on chemically equivalent atoms.

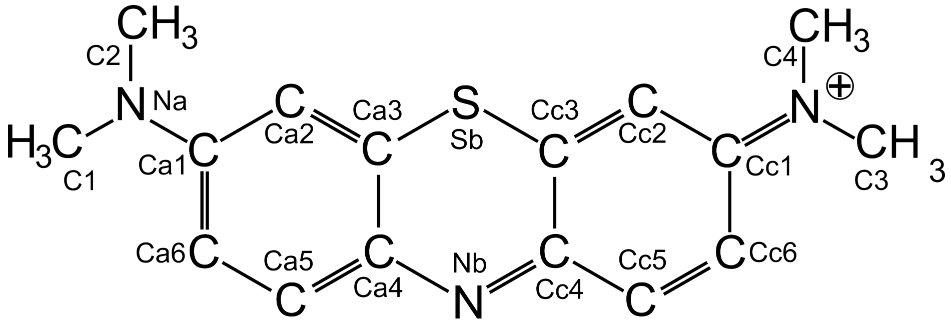

Figure S1: Structure and atom names of Methylene Blue. Not all hydrogens are shown, but their names take the name of the carbon atom plus an integer, *e.g.*, HA2 is attached to CA2 and H21, H22 and H23 are attached to carbon C2.

A single MeB molecule was geometry-optimized at the B97/3-21G level of DFT theory and placed at the center of a rectangular box that was filled with 2031 TIP3P water molecules[16]. A 1.0 nm cut-off was used for the Van der Waals' interactions, which were modeled with Lennard-Jones potentials, while the Coulomb interactions were computed with the smooth particle mesh Ewald method[21], using a 1.0 nm real space cut-off and a grid spacing of 0.12 nm and a relative

Table S1: Amber03 atomtypes and partial charges for Methylene Blue. Atom names are defined in Figure S1.

name	type	charge (e)
Ca1	CA	0.054312
Na	NA	0.154719
C1	CT	-0.284923
H11	H1	0.128201
H12	H1	0.128201
H13	H1	0.128201
C2	CT	-0.275632
H21	H1	0.127095
H22	H1	0.127095
H23	H1	0.127095
Ca2	CA	-0.205067
Ha2	HA	0.181067
Ca3	C	-0.101342
Ca4	CA	0.624779
Ca5	CA	-0.286158
Ha5	HA	0.187369
Ca6	CA	-0.141958
Ha6	HA	0.158598
Nb	NB	-0.712421
Sb	S	0.049117
Cc1	CA	0.054312
Nc	NA	0.154719
C3	CT	-0.284923
H31	H1	0.128201
H32	H1	0.128201
H33	H1	0.128201
C4	CT	-0.275632
H41	H1	0.127095
H42	H1	0.127095
H43	H1	0.127095
Cc2	CA	-0.205067
Hc2	HA	0.181067
Cc3	C	-0.101342
Cc4	CA	0.624779
Cc5	CA	-0.286158
Hc5	HA	0.187369
Cc6	CA	-0.141958
Hc6	HA	0.158598

tolerance at the real space cut-off of 10^{-5}. The simulation box, containing 6,131 atoms, was equilibrated for 1 ns at the force field level of theory. During this equilibration, the coordinates of the MeB atoms were kept fixed. The temperature was maintained at 300 K with the v-rescale thermostat[22], while the pressure was kept constant at 1 atmosphere using the Berendsen isotropic pressure coupling algorithm[23], with a time constant of 1 ps. The SETTLE algorithm was applied to constrain the internal degrees of freedom of water molecules[24], enabling a time step of 2 fs in the classical MD simulations.

After equilibration at the force field (MM) level, the system was further equilibrated at the QM/MM level for 10 ps. The time step was reduced to 1 fs. The Methylene Blue molecule was modelled at the DFT level, using the B97 functional[25], in combination with the 3-21G basis set[26]. The water solvent was modelled with the TIP3P force field[16]. The QM system experienced the Coulomb field of all MM atoms within a 1.0 nm cut-off sphere and Lennard-Jones interactions between MM and QM atoms were added. The singlet electronic excited state (S_1) was modeled with time-dependent DFT (TD-DFT)[27], using the B97 functional in combination with the 3-21G basis set for the QM region (*i.e.,* TD-B97/3-21G)[26]. At this level of QM/MM theory, the excitation energy is 2.5 eV. The overestimation of the vertical excitation energy is due to the limited accuracy of the employed level of theory, but it can be easily compensated by adding an off-set to the cavity resonance energy. The QM/MM simulations were performed with GROMACS version 4.5.3[28], interfaced to Gaussian16[29].

2.2 MeB-BSW system

From a QM/MM trajectory of MeB in the S_0 state, 1024 snapshots with different $S_0 \rightarrow S_1$ excitation energies were randomly selected as the initial configurations of $N = 1024$ molecules to account for inhomogeneous broadening around the absorption maximum at $E_{\text{exc}}^{\text{MeB}} = 2.50$ eV at the TD-B97/3-21G level of TDDFT theory. These molecules, including their solvent environment, were placed along the z-axis at equal intermolecular separations on the surface of a one-dimensional photonic crystal (Figure 1 in the main text) of width $L_z = 250\ \mu$m. The experimental dispersion of the Bloch

surface wave[9] was fitted with a linear function, $E_{\text{BSW}}(k_z) = a \cdot k_z + b$ with $a = 0.119$ eVμm^{-1} and $b = 0.771$ eV, and also shifted by 0.317 eV to provide a red-detuning of $\Delta = 341$ meV of the BSW energy at $k_z = 9$ μm^{-1} with respect to the absorption maximum $E_{\text{exc}}^{\text{MeB}} = 2.50$ eV of MeB. The resulting dispersion of the BSW is shown as the dashed-dotted line in Fugure S2**a**. The dispersion was modelled with 240 discrete modes, half of which are positive (*i.e.*, $k_{z,p} = 2\pi p/L_z$ with $358 \leq p \leq 477$) and half of which are negative (*i.e.*, $k_{z,p} = -2\pi p/L_z$ with $358 \leq p \leq 477$). To maximize the collective light-matter coupling strength, the transition dipole moments of all molecules were aligned to the vacuum field at the start of the simulation. With a vacuum field strength of $\mathbf{E}_y = 0.0000402473$ a.u. (0.207 MV cm^{-1}) the Rabi splitting, defined as the energy gap between the upper (UP) and lower polaritons (LP) at the wave vector where the molecular excitation energy matches the cavity mode energy, was $\hbar\Omega_{\text{R}} = 407$ meV. This value was chosen to provide a similar ratio between the disorder strength, $\sigma = 63$ meV, of MeB at the QM/MM level of theory and the Rabi splitting as in the experimental study[9] with the Rabi splitting of 142 meV and the disorder strength estimated to be $\sigma = 22$ meV as typical of J-aggregates[30,31]. The resulting BSW-polariton dispersion is depicted in Figure S2**a** with colors indicating the contribution of all BSW modes to each polariton state. In addition, the group velocities of the LP and UP branches are shown in Figure S2**b**.

All simulations were inititated with an off-resonant excitation into the first excited state, S_1, of the MeB molecule at the centre of the molecular chain, *i.e.*, at $z = 125$ μm. To analyse how polariton transport changes along the LP branch, partial wave functions, $|\Psi^{\text{part}}(z,t)\rangle$ (Equations 30, 31 and 33), were extracted from energy/wave vector windows of widths $\Delta E = 0.1$ eV and $\Delta k_z = 0.5$ μm^{-1} centred at different energies, E_c, and wave vectors, k_z^c, according to Table S2.

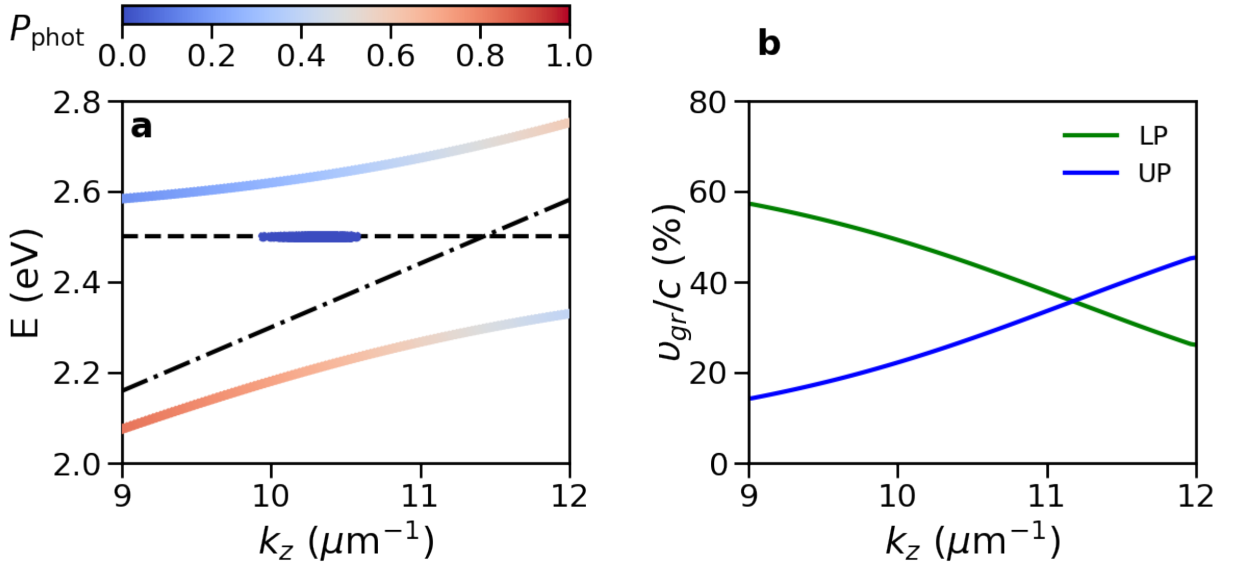

Figure S2: Panel **a**: Dispersion of BSW-polaritons. The total contribution of all cavity modes to each polaritonic state (P_{phot}) is indicated by colors. Dispersion of the BSW is shown as the dashed-dotted line, and the absorption maximum to the first excited state of Methylene Blue at the TD-B97/3-21G level of TDDFT theory is shown as the dashed line. Only the positive part of the dispersion (*i.e.* with $k_z > 0$) is shown. Panel **b**: Group velocity, $\upsilon_{\text{gr}} = \partial\omega_{\text{pol}}/\partial k_z$, of lower (LP, green line) and upper polaritons (UP, blue line) as a fraction of the speed of light, c.

Table S2: Ranges of energies (E, first column) and wave vectors (k_z, third column) at which partial wave functions, $|\Psi^{\text{part}}(z,t)\rangle$, were extracted. The second column indicates the energy at the centre of each energy/wave vector window. The fourth and fifth columns indicate, respectively, the wave vector of the polariton state corresponding to the centre of each window (k_z^c) and the total contribution of all BSW modes ($|\alpha_m^c|^2 = \sum_p^{n_{\text{modes}}} |\alpha_p^m|^2$) to this state.

E-range (eV)	E_c (eV)	k_z-range (μm^{-1})	k_z^c (μm^{-1})	$\lvert\alpha_m^c\rvert^2$
$[2.05; 2.15]$	2.10	$[9.00; 9.50]$	9.25	0.81
$[2.08; 2.18]$	2.13	$[9.25; 9.75]$	9.50	0.79
$[2.10; 2.20]$	2.15	$[9.50; 10.00]$	9.75	0.76
$[2.13; 2.23]$	2.18	$[9.75; 10.25]$	10.00	0.73
$[2.15; 2.25]$	2.20	$[10.00; 10.50]$	10.25	0.69
$[2.18; 2.28]$	2.23	$[10.25; 10.75]$	10.50	0.65
$[2.20; 2.30]$	2.25	$[10.50; 11.00]$	10.75	0.61
$[2.22; 2.32]$	2.27	$[10.75; 11.25]$	11.00	0.57
$[2.23; 2.33]$	2.28	$[11.00; 11.50]$	11.25	0.53

3 Analysis of the wave packets

3.1 Simulation of $N = 1024$ molecules

3.1.1 Partial wave packet

To extract how different lower polariton states contribute to the propagation of the total wavefunction, $|\Psi(z,t)\rangle$ (Equation 20), we constructed partial wave functions, $|\Psi_w^{\text{part}}(z,t)\rangle$ (Equations 30, 31 and 33), in windows w, representing fixed energy/wave vector intervals (Table S2). The time-evolution of the probability densities of the polaritonic states within these windows ($|\Psi_w^{\text{part}}(z,t)|^2$, Equation 33), is shown in Figure S3. In all cases, the partial wave packet formed by the states within the window propagates, indicating that LP states from all windows contribute to the overall transport.

To quantify how the transport regime of polaritons changes along the LP branch, we computed the mean squared displacements (MSD_w, Equation 34) of the partial wave functions associated with the windows (Figure S4), and fitted these MSD_w's to Equation 35 in order to extract the transport exponents, β (dashed lines in Figure S4). To avoid contamination of the MSD_w's by the noise that appears in regions beyond the distance defined by the group velocity of the lower polaritons, we included in the calculation of the MSD_w's only those points of the partial wave function, which correspond to coordinates lying within the cone bounded by the maximum group velocity of the LP states in each window (yellow lines in Figure S3).

In Figure 3**e** in the main text, the transport exponent, β, is plotted as a function of the total contribution of all BSW modes, $|\alpha_m^w|^2$, to the state ($|\psi_m^w\rangle$) whose wave vector is at the centre of the window w (Table S2). These plots suggest that the transport exponent increases with the photonic content as we move down in energy along the LP branch. The decrease in the transport exponent when the photonic content of a LP state decreases indicates a transition of the transport regime from ballistic motion at large photonic contents to diffusion at lower photonic contents, in line with experiments[9,32]. Furthermore, for lower polaritons in all windows, the transport exponent remains,

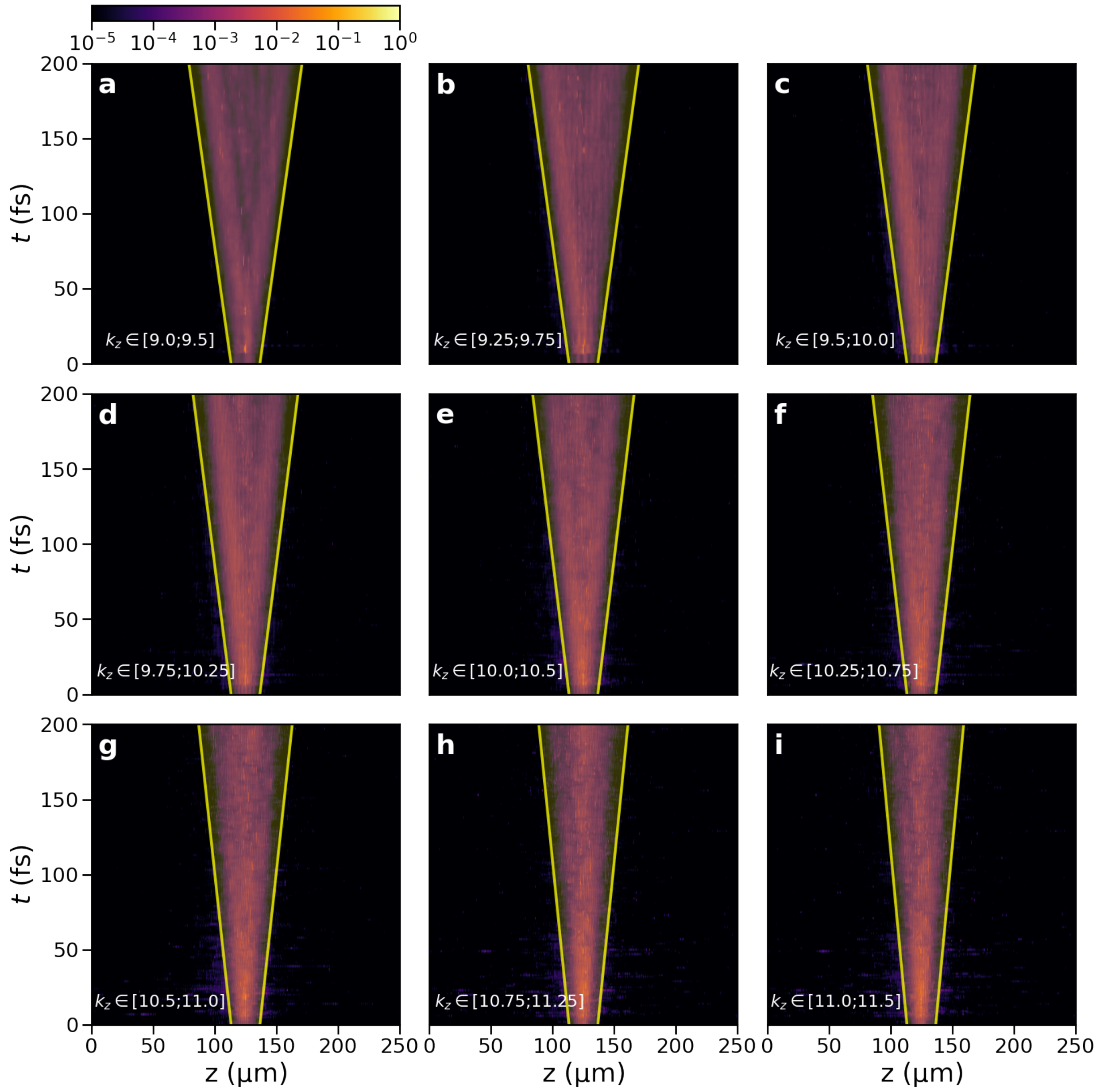

Figure S3: Time-space maps of the probability amplitude of the partial wave function $|\Psi_w^{\text{part}}|^2$ associated with different energy and wave vector windows (Table S2). The yellow lines represent the cone bounded by the maximum group velocity of lower polaritons in each window.

within the error, between one and two, with a crossover between the two transport regimes around a photonic content of $|\alpha_m|^2 \approx 0.7$.

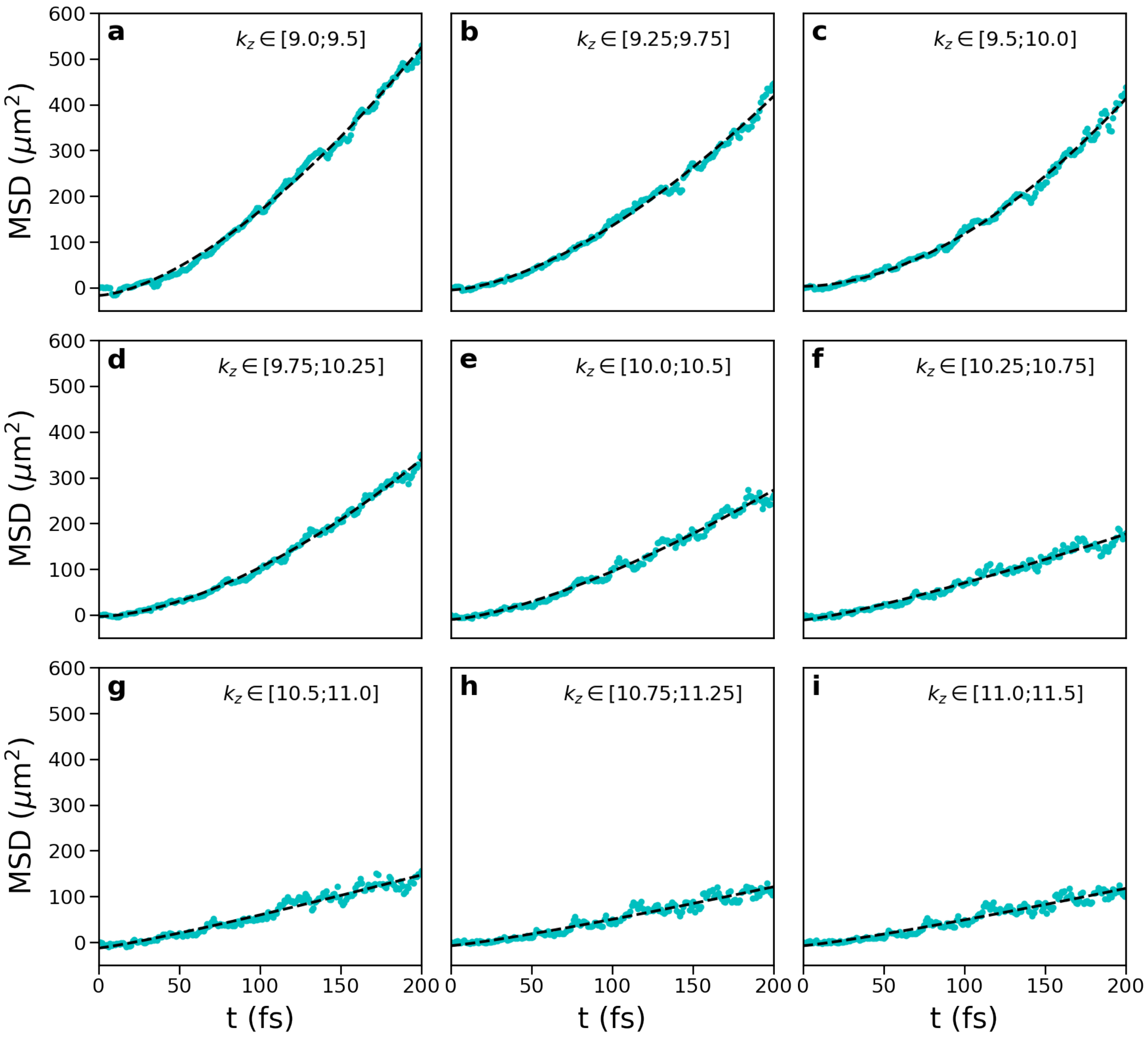

Figure S4: Mean squared displacement, $\text{MSD}_w(t) - \text{MSD}_w(0)$, of partial wave functions $|\Psi_{\text{part}}|^2$ as a function of time, extracted from different wave vector windows. Dashed lines are fits of the cyan dots to $D_\beta \cdot t^\beta$.

3.1.2 Estimation of transport velocities and diffusion coefficients

To provide a quantitative comparison between MD simulations and experiment, we extracted the transport velocity in the ballistic regime and the diffusion coefficient in the diffusion regime from the MSD_w of partial wave functions (Figure S4). In line with experiment[9], in the ballistic regime, the polariton states propagate ballistically with a velocity smaller than the group velocity of the lower polaritons, with the deviation decreasing at higher photonic contents, as demonstrated in Figure S5 (black dots and black line). Qualitatively, the deviation from the group velocity decreases from $\Delta v = 56$ μm ps^{-1} at $|\alpha_{\text{ph}}|^2 = 0.69$ to $\Delta v = 50$ μm ps^{-1} at $|\alpha_{\text{ph}}|^2 = 0.81$.

As we have shown in a previous work[15], the expansion of the polariton, and therefore the diffusion coefficient, is overestimated due to the small number of molecules in our simulations compared to the the realistic number of molecules on the order of $10^5 - 10^8$[33–36]. Indeed, the diffusion coefficient extracted from the MSD of the partial wave functions in the diffusion regime (red squares in Figure S5), is approximately one order of magnitude larger than in the experiment[9].

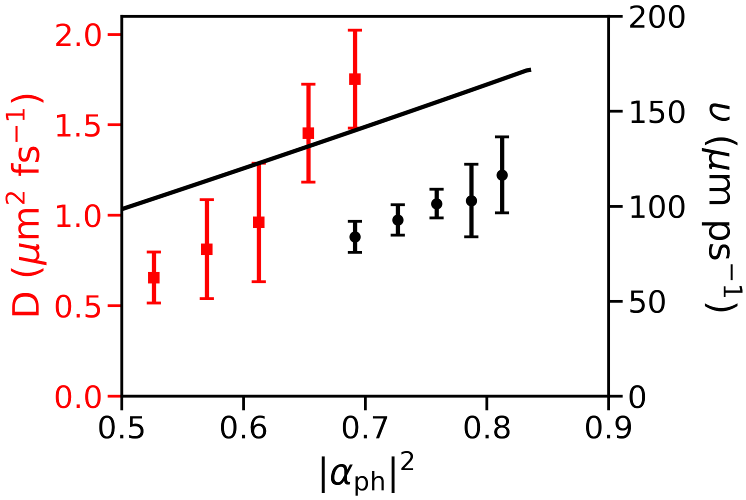

Figure S5: Diffusion coefficient (red squares) and ballistic expansion velocities (black circles) extracted from MD simulations. The black line depicts the group velocity of the LP branch (Figure S2**b**).

3.2 Simulation of two-level systems with static disorder

To understand whether the transition from ballistic to diffusive transport along the lower polariton branch is caused by population exchanges between polariton states due to nonadiabatic coupling or whether it can be solely explained by excitation energy disorder, we performed simulations of $N = 1000$ two-level systems with static disorder. In these simulations, the excitation energies of the two-level systems were drawn from a Gaussian distribution (Equation 9 in the main text). Figures S6, S7 and S8 depict the time-evolution of the partial wave functions within different energy and wave vector windows (Table S2) in simulations with the static energy disorder strengths of $\sigma = 22$ meV, 63 meV, and 100 meV, respectively. In Figure S9**a**-**c**, the corresponding MSD$_w$'s are plotted. The disorder strengths of $\sigma = 22$ meV and 63 meV correspond to typical linewidths of the absorption spectra of TDBC J-aggregates[30,31] and of Methylene Blue at the TD-DFT/B97//3-21G level of theory in our MD simulations, and the disorder strength of $\sigma = 100$ meV was selected to provide a comparison with highly disordered systems, in which polaritons may experience partial localisation.

The plots of the MSD$_w$'s are very well fitted with Expression 35 (see Table S3 for the values of a regression coefficient), allowing for an accurate estimation of the transport exponent β. As demonstrated in Figure S9**d**-**f**, the variation of β with respect to the photonic content, $|\alpha_m|^2$ of the polaritonic states strongly depends on the strength of the disorder. For the smallest disorder ($\sigma = 22$ meV, Figure S9**d**), the transport exponent, within the error, remains close to two within the whole range of considered wave vectors, which implies no transition to diffusion. For a larger disorder ($\sigma = 63$ meV, Figure S9**e**), the transport exponent is reduced with decreasing $|\alpha_m|^2$, yet the transition to diffusion is incomplete as β remains larger than one even in the energy window with the LP states with the smallest photonic contribution. In contrast, the transition appears to be complete in the case of the largest disorder ($\sigma = 100$ meV, Figure S9**f**), for which the transport exponent reaches, within the error, the value of one already at $|\alpha_m|^2 \approx 0.7$.

To explore whether the transition to diffusive transport occurs on a timescale longer than 200 fs in the case of a moderate disorder, we repeated simulations with $\sigma = 63$ meV but for a

longer time ($t = 1$ ps) with the following parameters: $N = 1600$, $n_{\text{modes}} = 384$, $L_z = 400\ \mu$m. Figures S10 and S11**a-b** show the propagation of the partial wave function within different energy and wave vector windows and the corresponding mean squared displacement in this simulation. The extracted transport exponent displays a similar trend with the photonic content in the case of a short simulation ($t \in [0; t_f]$ with $t_f = 200$ fs (Figure S11**c**, black circles) and $t_f = 400$ fs (Figure S11**c**, green triangles)) and a long simulation ($t \in [t_s; 1000\text{ fs}]$ with $t_s = 0$ fs (Figure S11**d**, black circles), $t_s = 200$ fs (Figure S11**d**, blue diamonds) and $t_s = 400$ fs (Figure S11**d**, magenta squares)). However, the value of β is smaller on a longer timescale and becomes less than one for more excitonic-like LP states, which are closer in energy to (and can even overlap with) the molecular absorption spectrum and hence are more susceptible to the influence of disorder.

Table S3: Coefficients of determination (R^2) for the fits to Expression 35 of the MSD$_w$'s obtained for the partial wave function, $|\Psi_w^{\text{part}}(z,t)\rangle$, in static simulations of two-level systems with disorder strength σ of 22 meV (second column), 63 meV (third column), and 100 meV (forth column). The first column indicates ranges of wave vectors (k_z) at which partial wave functions were extracted.

k_z-range (μm^{-1})	R^2 ($\sigma = 22$ meV)	R^2 ($\sigma = 63$ meV)	R^2 ($\sigma = 100$ meV)
$[9.00; 9.50]$	1.0000	1.0000	0.9998
$[9.25; 9.75]$	1.0000	1.0000	0.9998
$[9.50; 10.00]$	1.0000	1.0000	0.9998
$[9.75; 10.25]$	1.0000	1.0000	0.9994
$[10.00; 10.50]$	1.0000	1.0000	0.9988
$[10.25; 10.75]$	1.0000	0.9999	0.9981
$[10.50; 11.00]$	1.0000	0.9999	0.9975
$[10.75; 11.25]$	1.0000	0.9998	0.9978
$[11.00; 11.50]$	1.0000	0.9996	0.9990

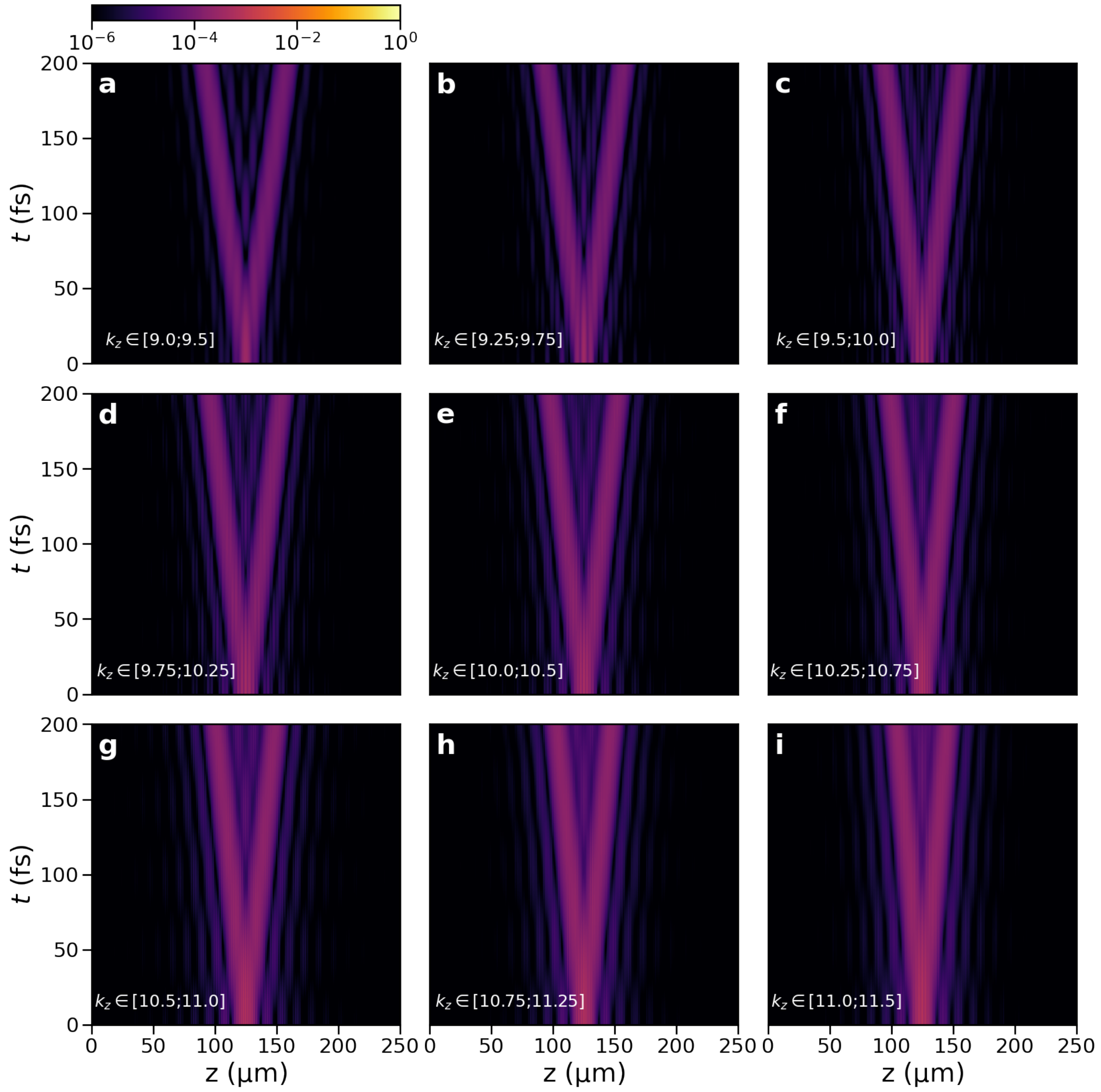

Figure S6: Time-space maps of the probability amplitude of the partial wave function, $|\Psi_w^{\text{part}}|^2$, associated with different energy and wave vector windows (Table S2) in simulations of static two-level systems with the excitation energy disorder $\sigma = 22$ meV.

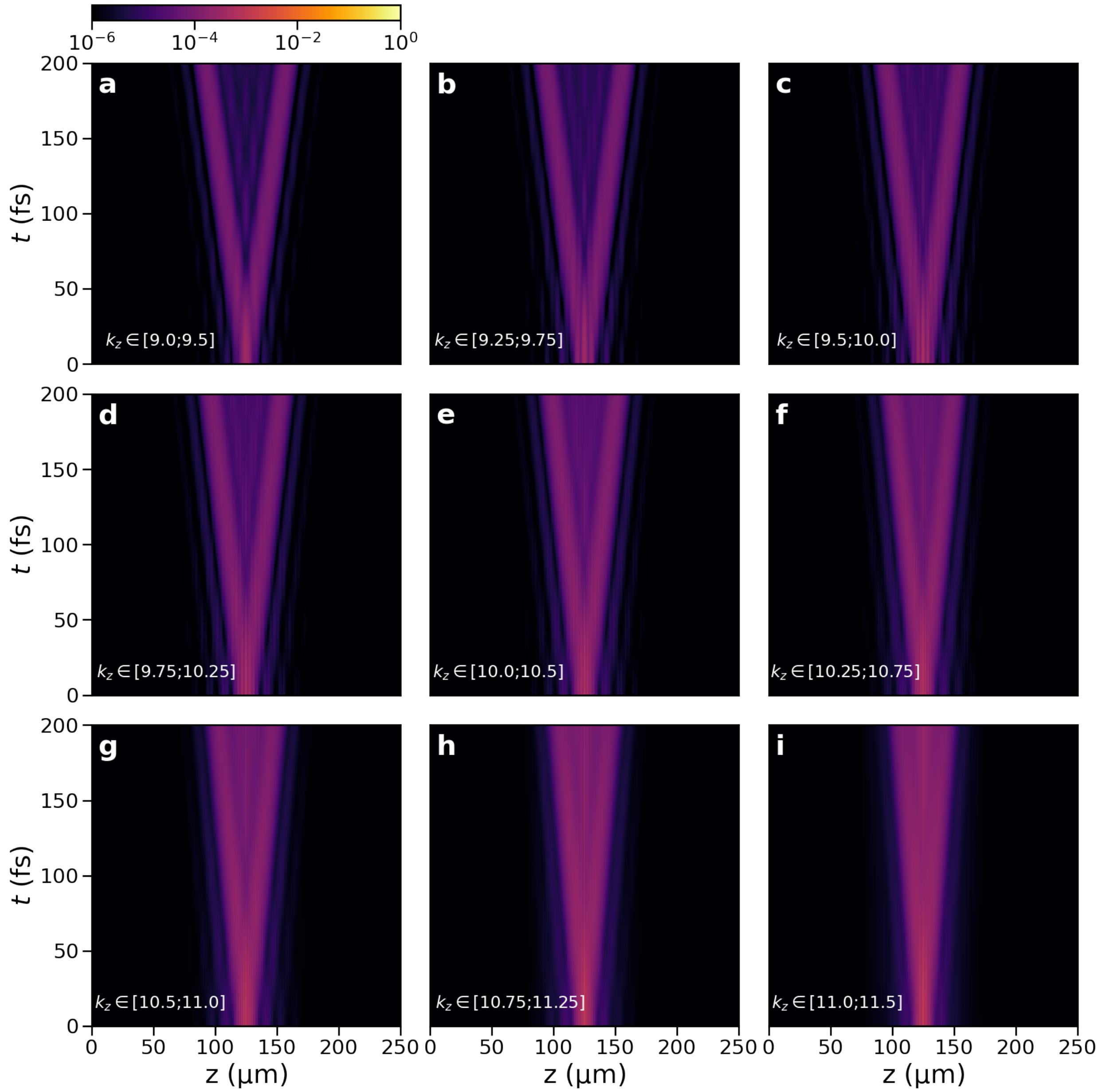

Figure S7: Time-space maps of the probability amplitude of the partial wave function, $|\Psi_w^{\mathrm{part}}|^2$, associated with different energy and wave vector windows (Table S2) in simulations of static two-level systems with the excitation energy disorder $\sigma = 63$ meV.

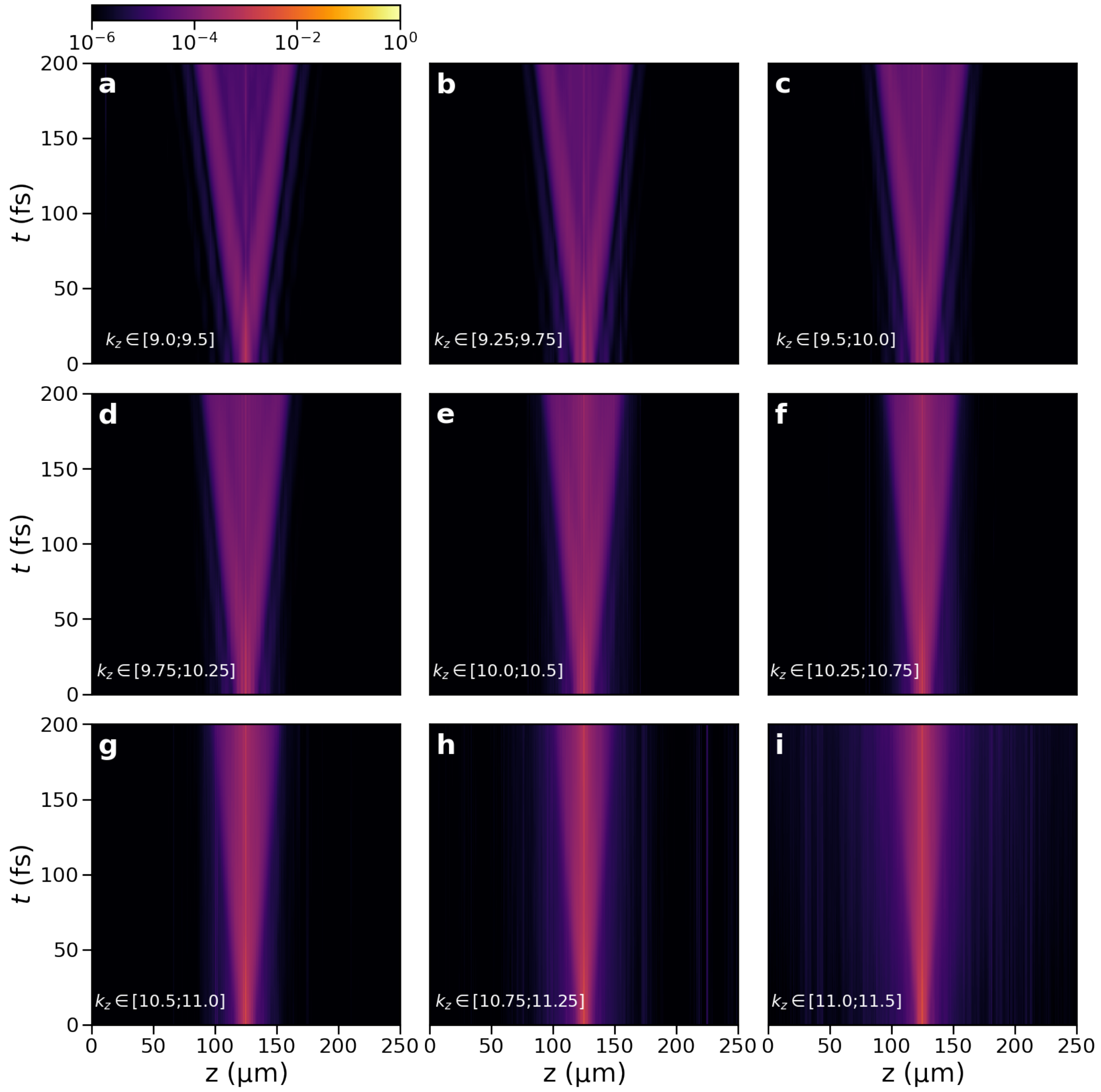

Figure S8: Time-space maps of the probability amplitude of the partial wave function, $|\Psi_w^{\text{part}}|^2$, associated with different energy and wave vector windows (Table S2) in simulations of static two-level systems with the excitation energy disorder $\sigma = 100$ meV.

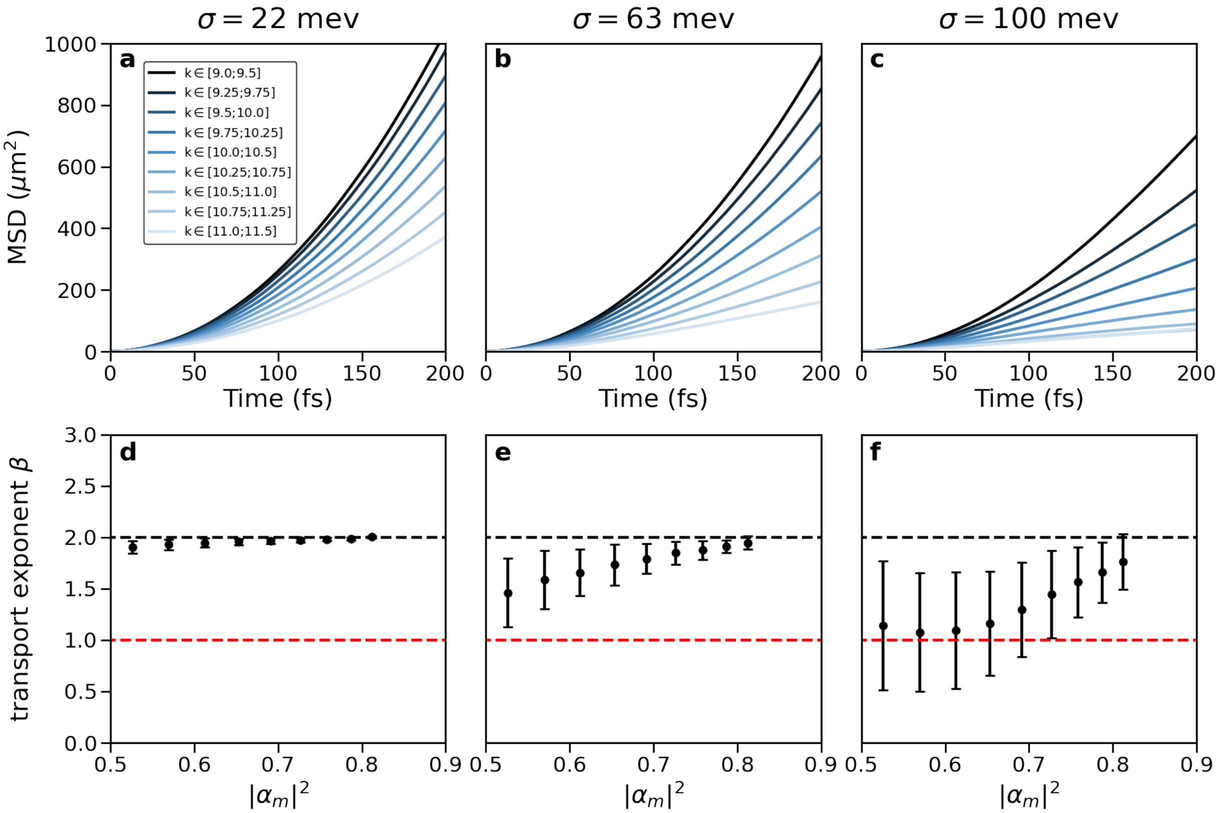

Figure S9: Panels **a**, **b** and **c**: Mean squared displacement (MSD_w) of the partial wave function, $|\Psi_w^{\mathrm{part}}|^2$, extracted from different wave vector windows in static simulations of two-level systems with the excitation energy disorder of $\sigma = 22$ meV (**a**), $\sigma = 63$ meV (**b**), and $\sigma = 100$ meV (**c**). Panels **d**, **e** and **f**: Values of the transport exponent, β, as a function of the BSW modes contribution $|\alpha_{\mathrm{m}}|^2$ to polaritonic states. The errors are standard deviations of five hundred individual runs.

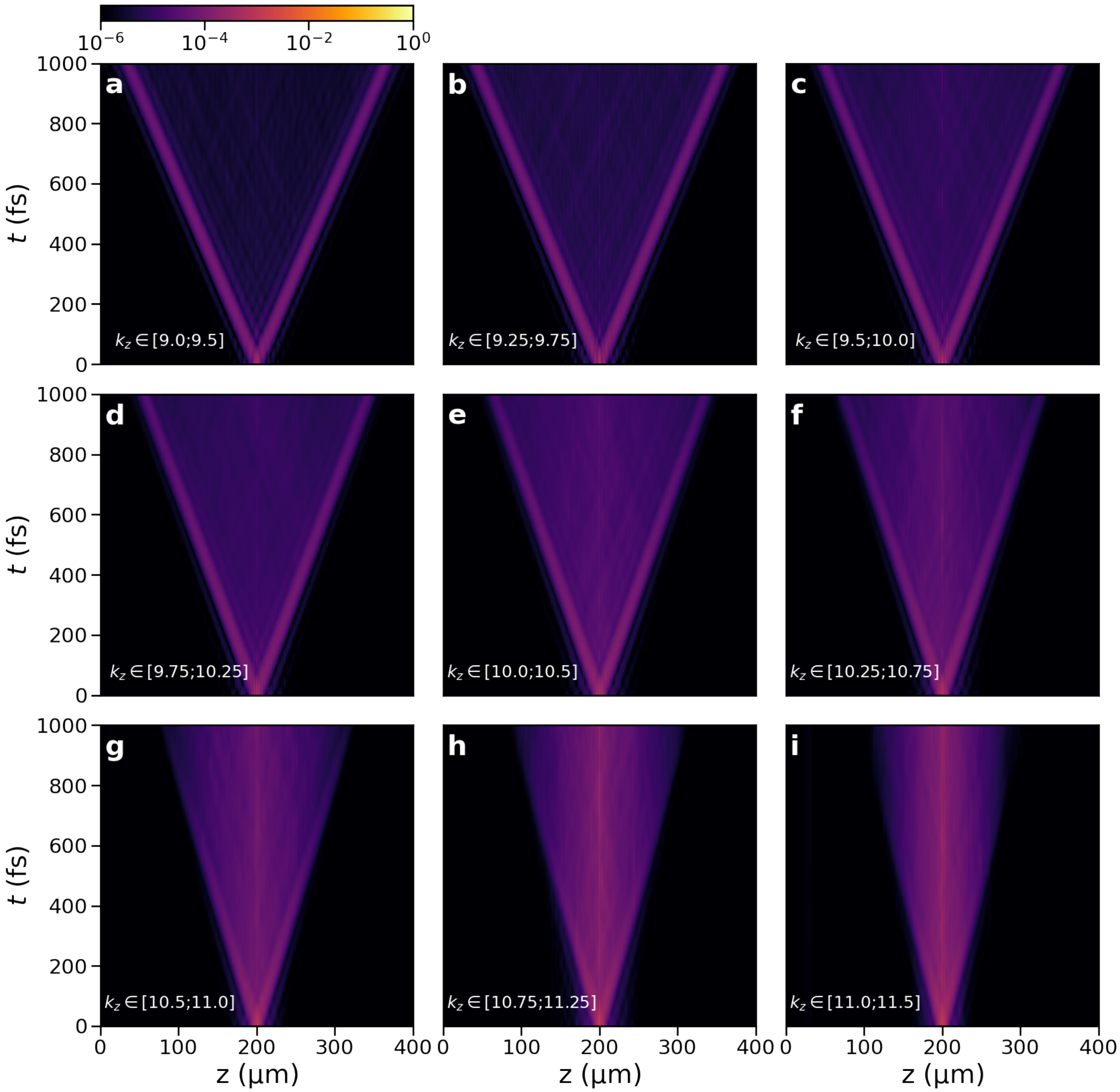

Figure S10: Time-space maps of the probability amplitude of the partial wave function, $|\Psi_w^{\mathrm{part}}|^2$, associated with different energy and wave vector windows (Table S2) in a picosecond-long simulation of static two-level systems with the excitation energy disorder $\sigma = 63$ meV.

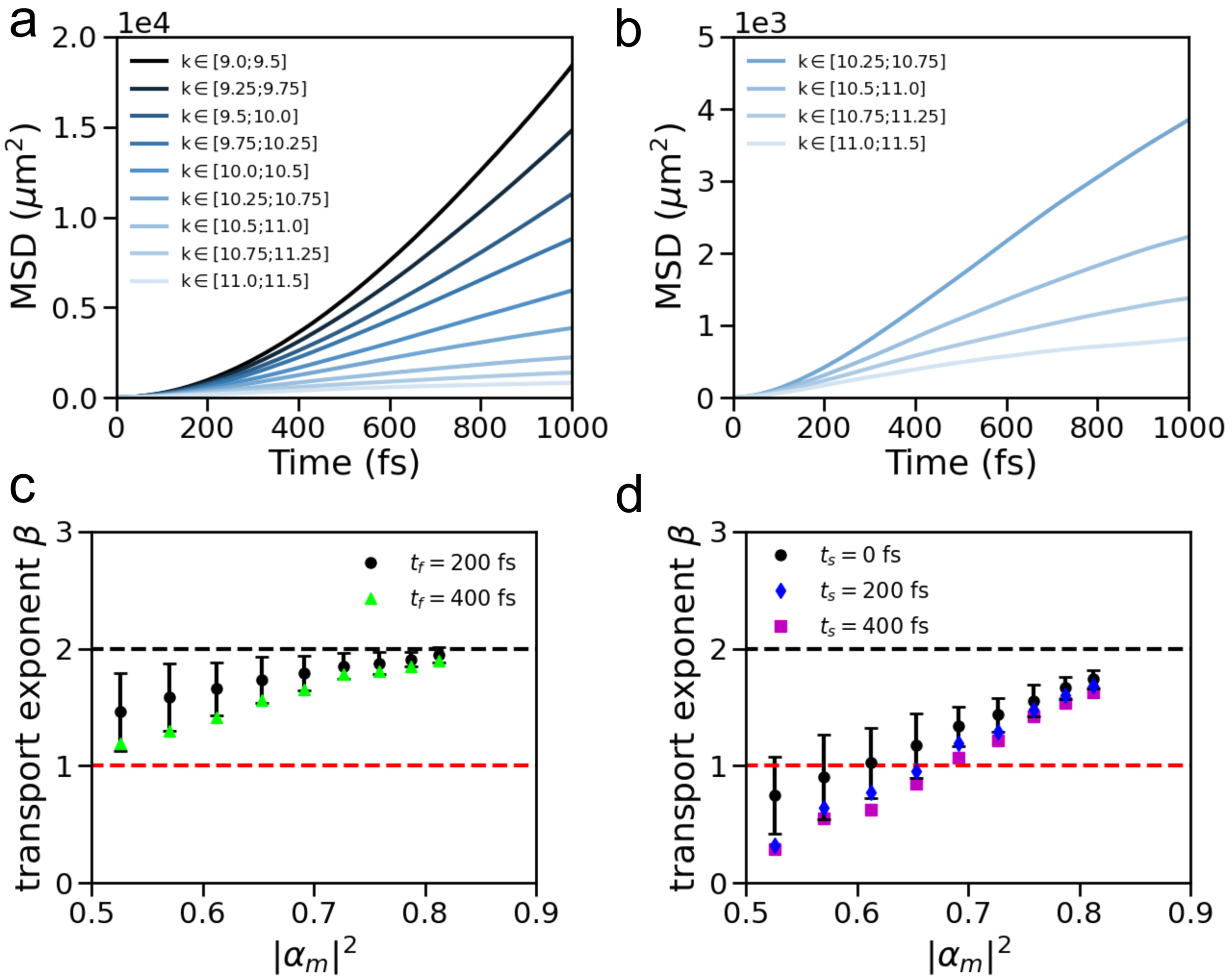

Figure S11: Panels **a**: Mean squared displacement (MSD_w) of the partial wave function, $|\Psi_w^{\mathrm{part}}|^2$, extracted from different wave vector windows in a picosecond-long static simulation of two-level systems with the excitation energy disorder of $\sigma = 63$ meV. Panel **b**: Same plots as in panel **a** but in a smaller scale and only of the MSD_w's for the four highest-energy windows. Panels **c** and **d**: Values of the transport exponent, β, as a function of the BSW modes contribution $|\alpha_\mathrm{m}|^2$ to polaritonic states extracted from the fit of the MSD_w to Equation 35 within time range $t \in [0; 200]$ fs (panel **c**, black circles), $t \in [0; 400]$ fs (panel **c**, green triangles), $t \in [0; 1000]$ fs (panel **d**, black circles), $t \in [200; 1000]$ fs (panel **d**, blue diamonds), and $t \in [400; 1000]$ fs (panel **d**, magenta squares). The error bars depict standard deviations of five hundred individual runs.

3.3 MD simulations with constraints on bond lengths and out-of-plane motions of Methylene Blue molecules

3.3.1 $\hbar\Omega_{\mathbf{R}} = 407$ meV

As we discuss in the main text, molecular vibrations are crucial for the observed crossover between the two regimes of polariton transport, because this crossover is caused by population exchange between bright and dark states, that is driven by nonadiabatic coupling. To provide further support for this conclusion, we also performed simulations with a reduced number of degrees of freedom. Specifically, we computed trajectories of $N = 1024$ Methylene Blue molecules in vacuum, with constraints on all bond lengths,[37] as well as on the out-of-plane motions of the heavy (carbon, nitrogen, oxygen and sulfur) atoms. Under these conditions, the excitation energy disorder strength was reduced from $\sigma = 63$ meV to $\sigma = 28$ meV.

Figure S12**a** depicts the contributions of the MeB excitations and of the BSW modes to the total wave function $|\Psi(t)|^2$ as a function of time. Throughout the simulation, the excitonic contribution remains above 90%, which implies that the imposed restrictions result in a much more modest population transfer between bright and dark states with the majority of the population remaining in the dark states, which are mostly composed of molecular excitons. The lower population transfer dynamics reflects a reduction of the nonadiabatic coupling[38] via (i) a suppression of the molecular motions, and (ii) a more narrow energy distribution of the dark states manifold. As the non-adiabatic coupling is defined as the product between the non-adiabatic coupling vector and the molecular displacements, reducing the latter decreases the population transfer. Likewise, since the non-adiabatic coupling vector for population transfer between dark and bright states is inversely proportional to the energy gap between these states[39], narrowing the distribution of the dark states increases that gap and thus also decreases the non-adiabatic population transfer.

Suppression of population exchange between bright and dark states leads to ballistic transport of all states in the LP branch. This is evident from the plots of the probability amplitudes of the partial wave functions $|\Psi_w^{\text{part}}(z,t)|^2$ associated with the different wave vector windows in Figure S13. In

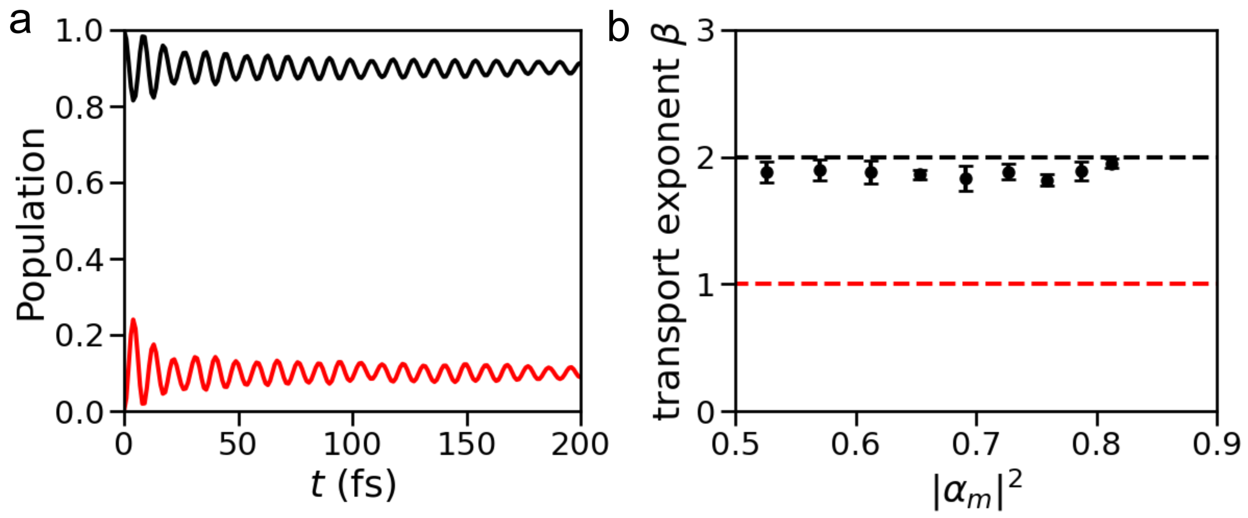

Figure S12: Panel **a**: Contribution of MeB excitations (black line) and BSW modes (red line) to the total wave function, $\Psi(z,t)$, in Molecular Dynamics simulations with constraints on bond lengths and out-of-plane motions. Panel **b**: The transport exponent β as a function of the BSW modes contribution $|\alpha_{\mathrm{m}}|^2$ to polaritonic states. The error bars are standard deviations of five runs.

all windows, the population propagates nearly with the maximal group velocity of that window (cyan lines in Figure S13). Accordingly, the plots of the MSD_w in Figure S14 display a quadratic time-dependence for all wave vector windows. Fitting Equation 35 to these MSD_w's reveals that the transport coefficients are always close to two (Figure S12**b**), suggesting ballistic motion that is independent of the photonic content of the LP states.

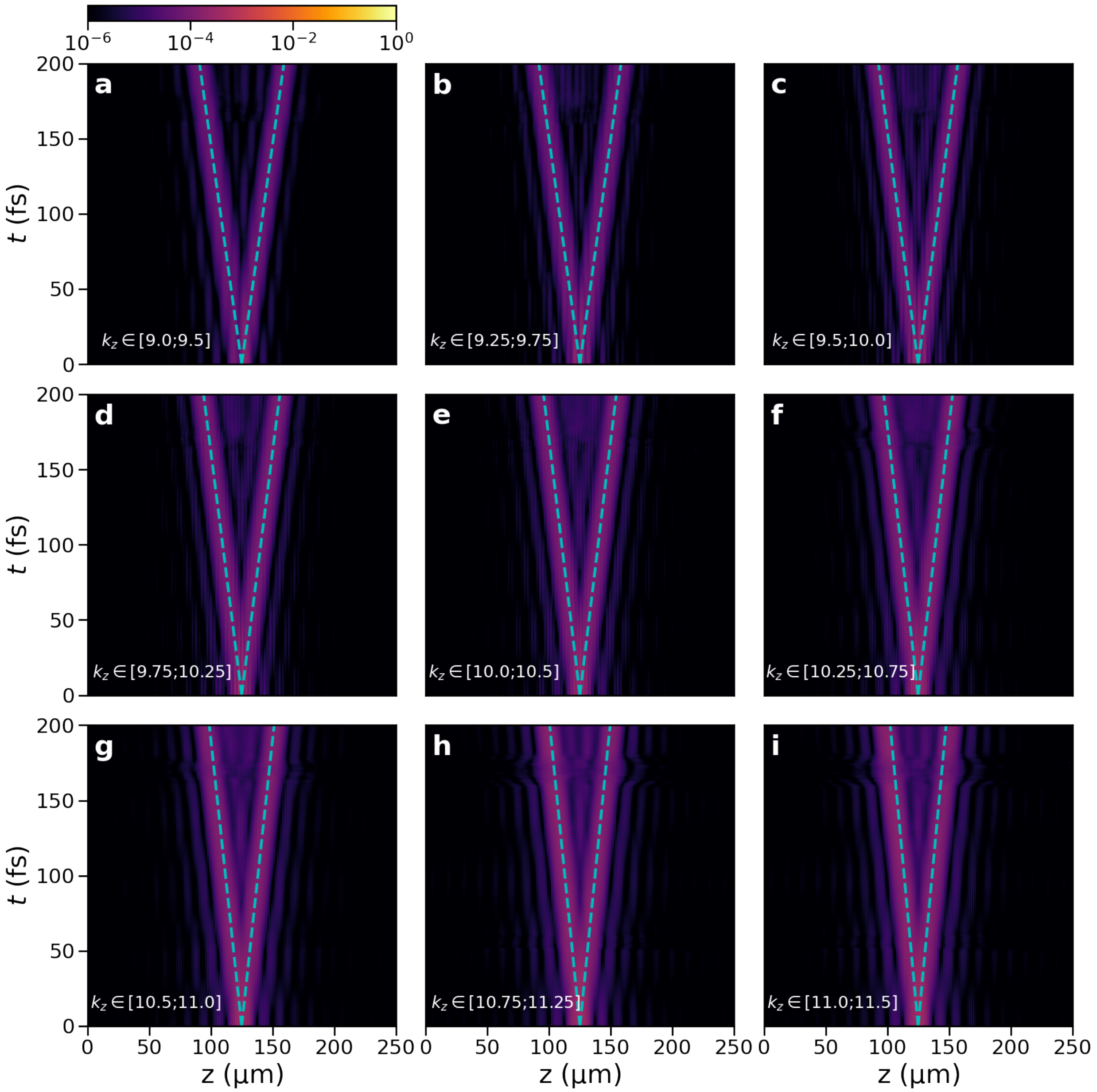

Figure S13: Time-space maps of the probability amplitude of the partial wave function $|\Psi^{\mathrm{part}}(z,t)|^2$ extracted through different energy and wave vector windows in simulations with constraints imposed on the nuclear degrees of freedom and $\hbar\Omega_{\mathrm{R}} = 407$ meV. Cyan lines correspond to the highest group velocity of LP in each window.

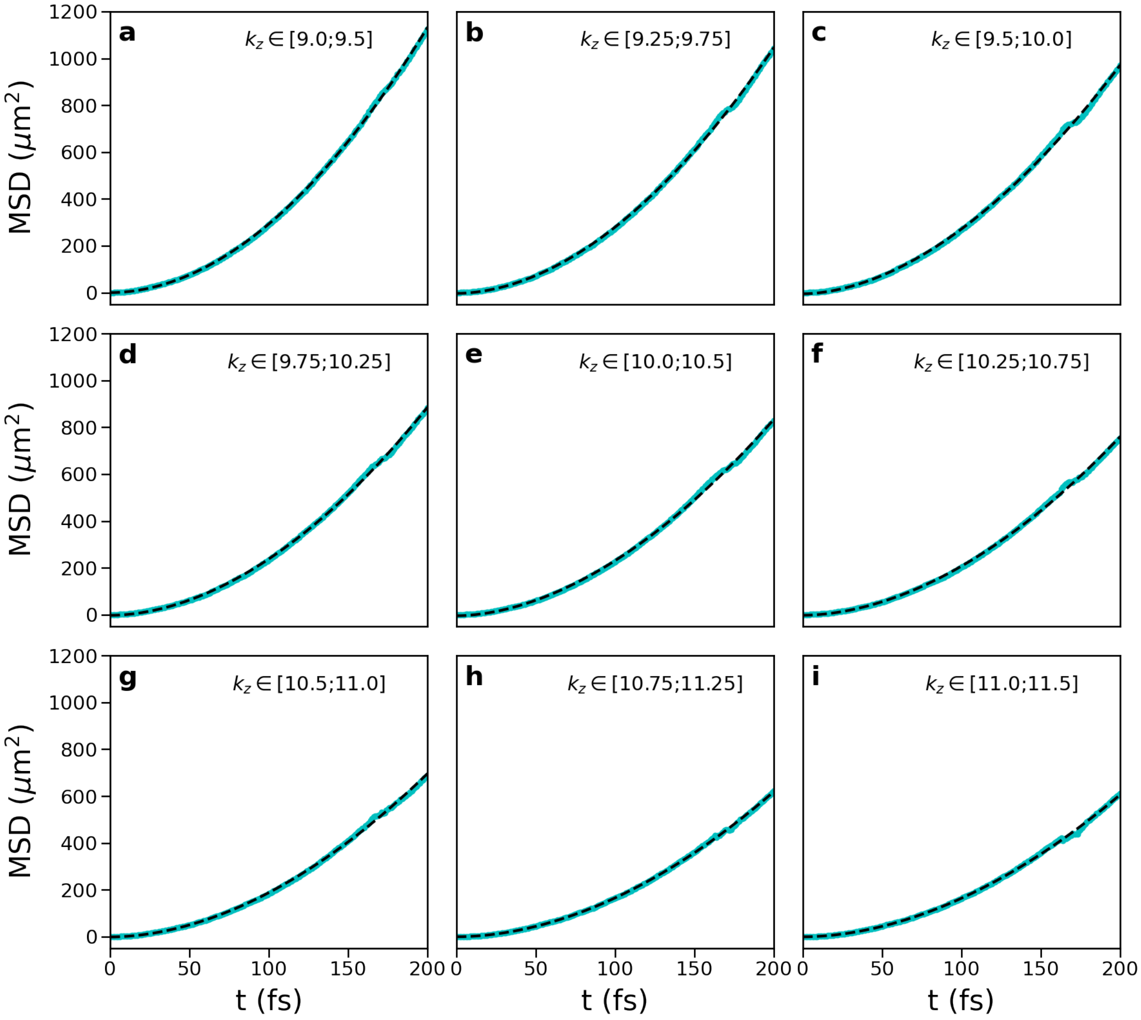

Figure S14: Mean squared displacement, $\text{MSD}_w(t) - \text{MSD}_w(0)$, of partial wave functions $|\Psi_w^{\text{part}}|^2$ as a function of time, extracted from different wave vector windows in simulations with constraints on bond lengths and out-of-plane motions and $\hbar\Omega_{\text{R}} = 407$ meV. Dashed lines are fits to $D_\beta \cdot t^\beta$.

3.3.2 $\hbar\Omega_\mathbf{R} = 181$ meV

To rule out the possibility that the absence of crossover in simulations with constraints on bond lengths and out-of-plane motions of MeB and at a Rabi splitting of $\hbar\Omega_\mathrm{R} = 407$ meV was due to the lack of overlap between dark states and lower polariton states, we repeated these simulations at a lowered Rabi splitting of $\hbar\Omega_\mathrm{R} = 181$ meV. With this value of the Rabi splitting, the ratio between molecular disorder and the collective coupling strength, $\sigma/g\sqrt{N} = 0.31$, was the same as in simulations with $\hbar\Omega_\mathrm{R} = 407$ meV and no constraint on molecular motions.

First, we performed simulations of static two-level systems whose energies were randomly sampled from a Gaussian distribution with $\sigma = 28$ meV (Equation 9 in the main text). The time propagation of the partial wave functions $|\Psi_w^\mathrm{part}(z,t)|^2$, shown in Figure S15, was ballistic in all energy windows, suggesting no transition to diffusion. This was further confirmed by extracting the transport exponent (Figure S17**a**), which remained close to two within the error for all lower polariton states. We note that in the two highest energy windows, the average transport exponent was reduced due to the overlap between polaritonic states and the molecular energy distribution, which resulted in back-scattering of the polariton states. However, this effect was not sufficient to render the transition from ballistic transport to diffusion complete, as was also the case in simulations with $\sigma = 63$ meV and $\hbar\Omega_\mathrm{R} = 407$ meV (Section 3.2) with the same ratio between the disorder strength and the collective coupling strength as here.

Next, we performed MD simulations of $N = 1024$ MeB molecules with constraints on bond lengths and out-of-plane motions of the heavy atoms, in a cavity with the same parameters as in the static simulations. The time-space maps of the probability amplitude of the partial wave function ($|\Psi_w^\mathrm{part}(z,t)|^2$, Figure S16) and the extracted values of the transport exponent (Figure S17**b**), although noisier, are consistent with those obtained in simulations of static two-level systems and demonstrate no clear transition to the diffusive regime of polariton propagation. This finding allows us to conclude that, even with a significant (but not very large), overlap between lower polaritons and dark states as was the case in experiment[9], the transition between the two polariton transport

regimes cannot be captured without the inclusion of molecular vibrations, which makes it possible for reversible population transfers between propagating bright states and stationary dark states.

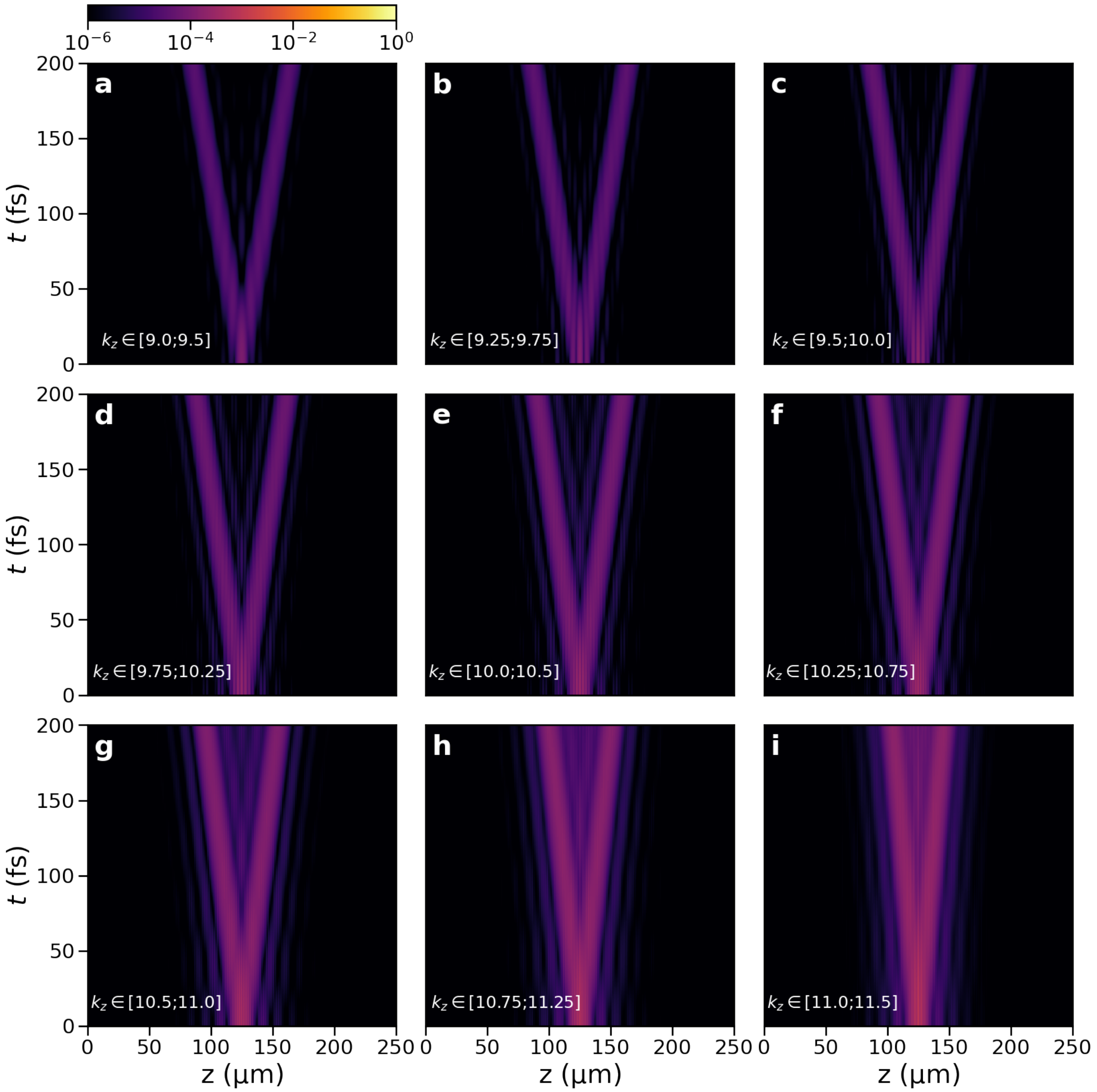

Figure S15: Time-space maps of the probability amplitude of the partial wave function $|\Psi^{\text{part}}(z,t)|^2$ associated with different energy and wave vector windows in simulations of static two-level systems with the excitation energy disorder $\sigma = 28$ meV and $\hbar\Omega_{\text{R}} = 181$ meV.

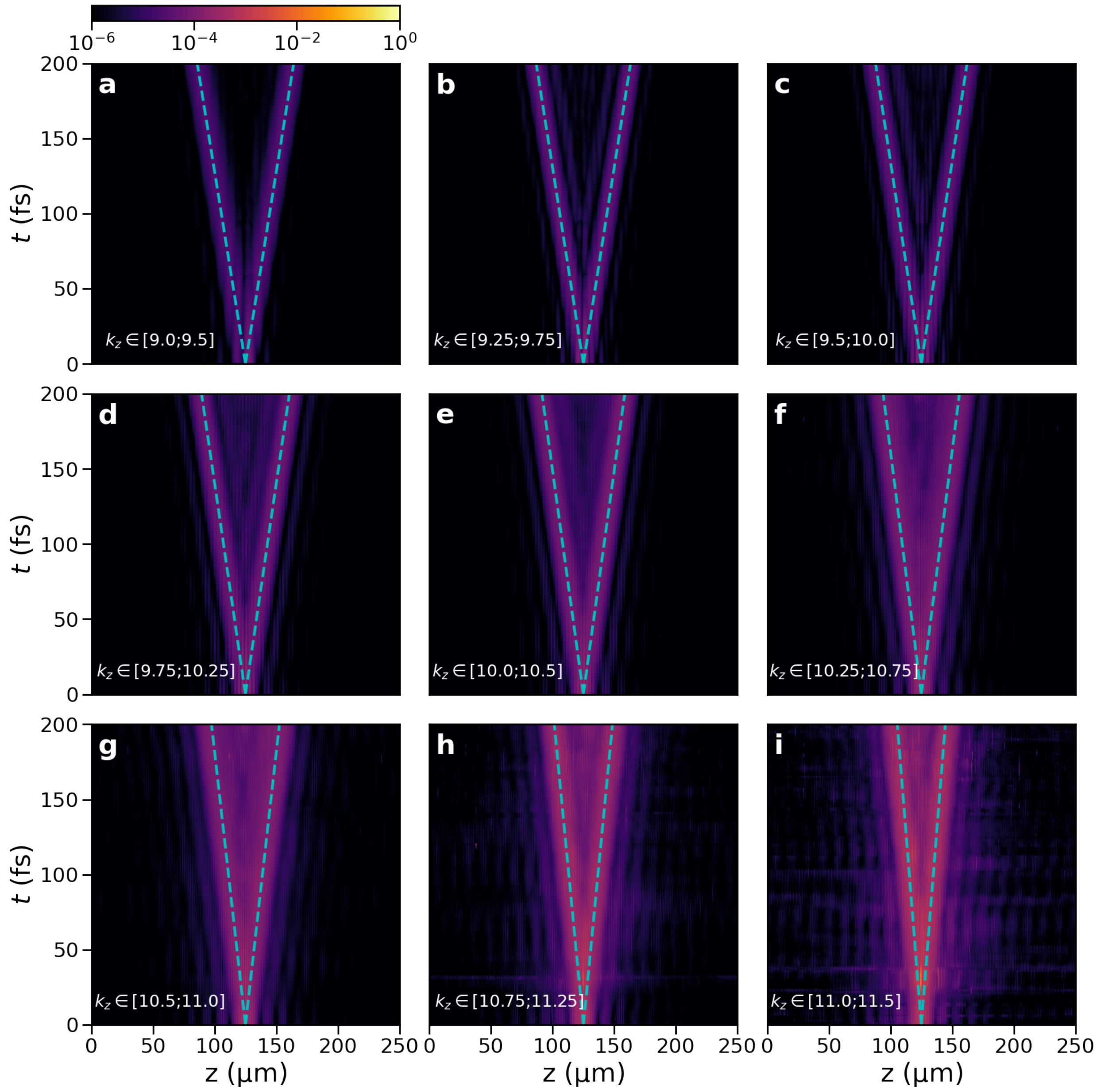

Figure S16: Time-space maps of the probability amplitude of the partial wave function $|\Psi^{\text{part}}(z,t)|^2$ extracted through different energy and wave vector windows in simulations with constraints imposed on the nuclear degrees of freedom and $\hbar\Omega_{\text{R}} = 181$ meV. Cyan lines correspond to the highest group velocity of LP in each window.

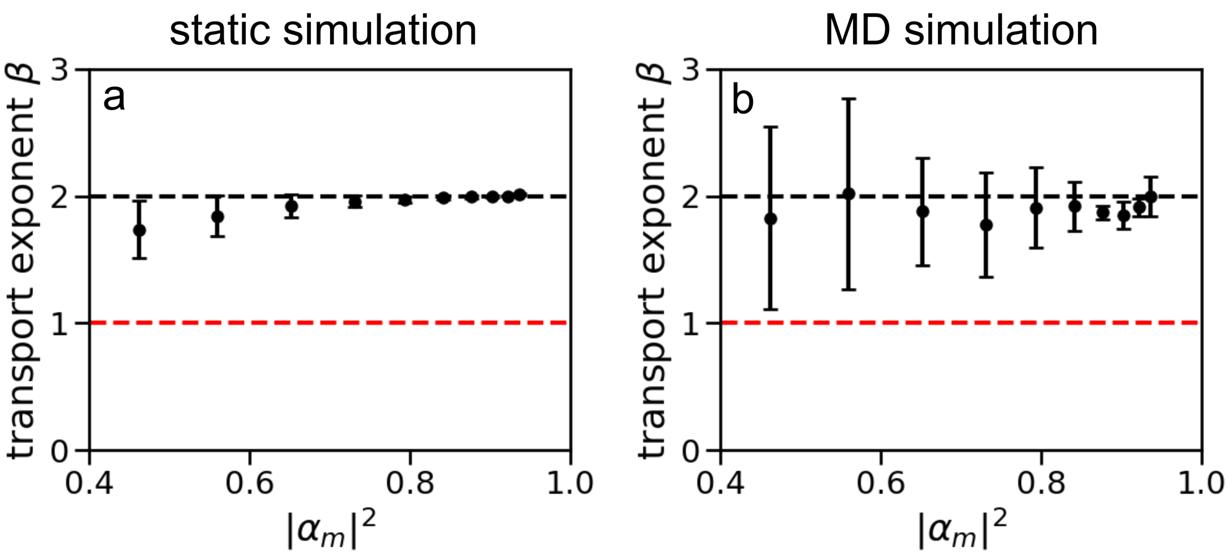

Figure S17: The transport exponent β as a function of the BSW modes contribution $|\alpha_{\mathrm{m}}|^2$ to polaritonic states in static simulations with $\sigma = 28$ meV (panel **a**) and MD simulations with constraints imposed on molecular motions (panel **b**) and with a Rabi splitting of $\hbar\Omega_{\mathrm{R}} = 181$ meV. The error bars are standard deviations of, respectively, five hundred and five runs.

3.4 Simulation of two-level systems with quasi-dynamic disorder

To further confirm that the observed crossover in the transport regime is not entirely due to structural disorder, we performed simulations of two-level systems with quasi-dynamic excitation energy disorder. In these simulations, excitation energies of the two-level systems were drawn from a Gaussian distribution (Equation 9 in the main text) and resampled with the time intervals drawn from a Poisson distribution[40] (Equation 10 in the main text). In Figure S18 and S19, the propagation of the partial wave function, $|\Psi_w^{\text{part}}|^2$, in simulations with a disorder strength of $\sigma = 63$ meV and with the average resampling time $\langle\tau\rangle$ of 5 fs and 10 fs, respectively, is plotted as a function of time and distance. Figure S20**a**-**b** shows the respective mean squared displacements. By fitting these MSD_w's with Expression 35, we extracted the values of the transport exponent, which are plotted in Figure S20**c**-**d** as a function of the photonic Hopfield coefficients. Similar to the model with static disorder, the quasi-dynamic simulations do not yield a complete transition from ballistic transport to diffusion, thus further underlying the role of vibrationally driven non-adiabatic population transfers in turning the propagation of polaritons with high excitonic content into a diffusion process.

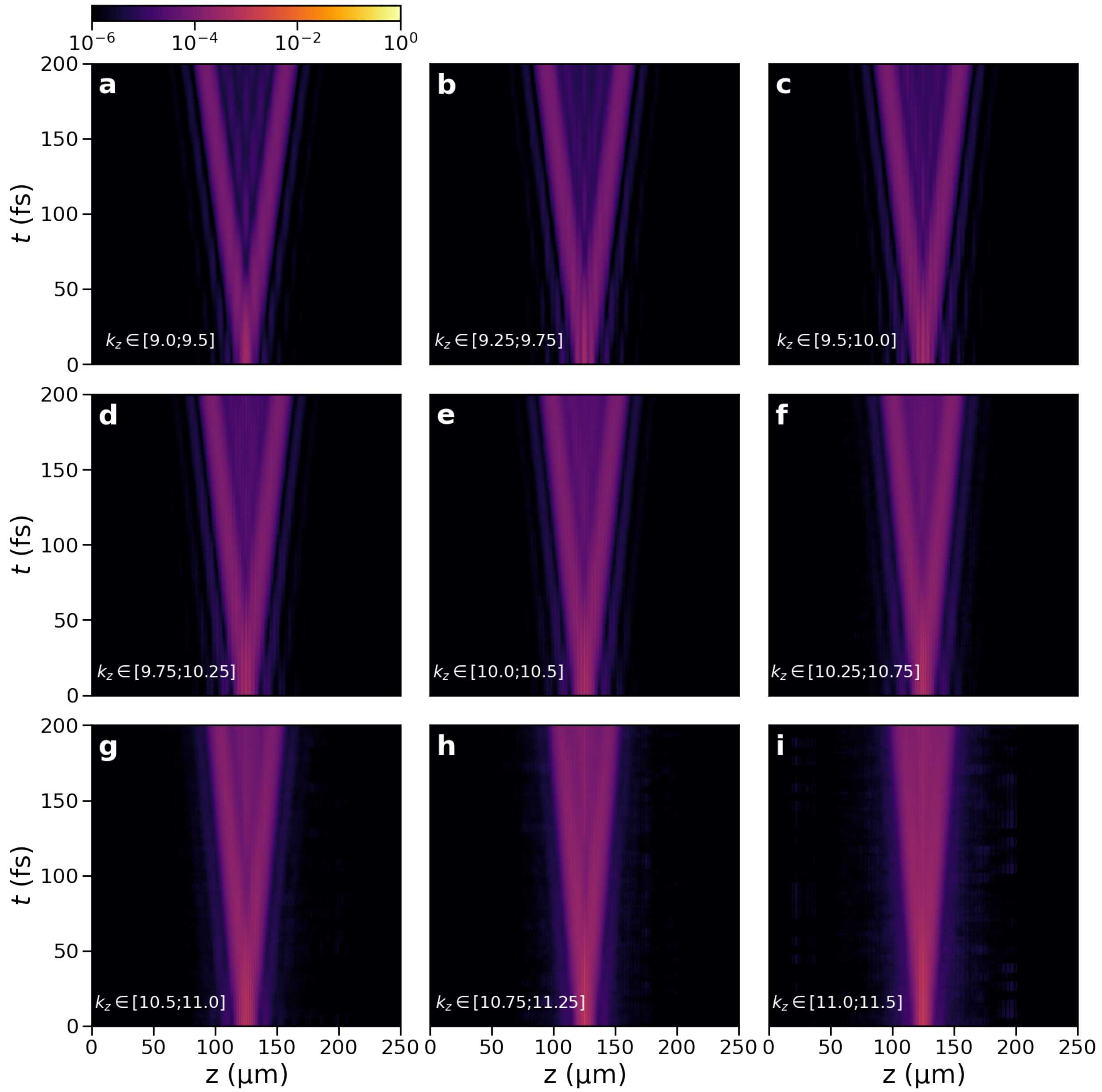

Figure S18: Time-space maps of the probability amplitude of the partial wave function, $|\Psi_w^{\text{part}}|^2$, associated with different energy and wave vector windows (Table S2) in quasi-dynamic simulations of two-level systems with the excitation energy disorder $\sigma = 63$ meV and average resampling time $\langle\tau\rangle = 5$ fs.

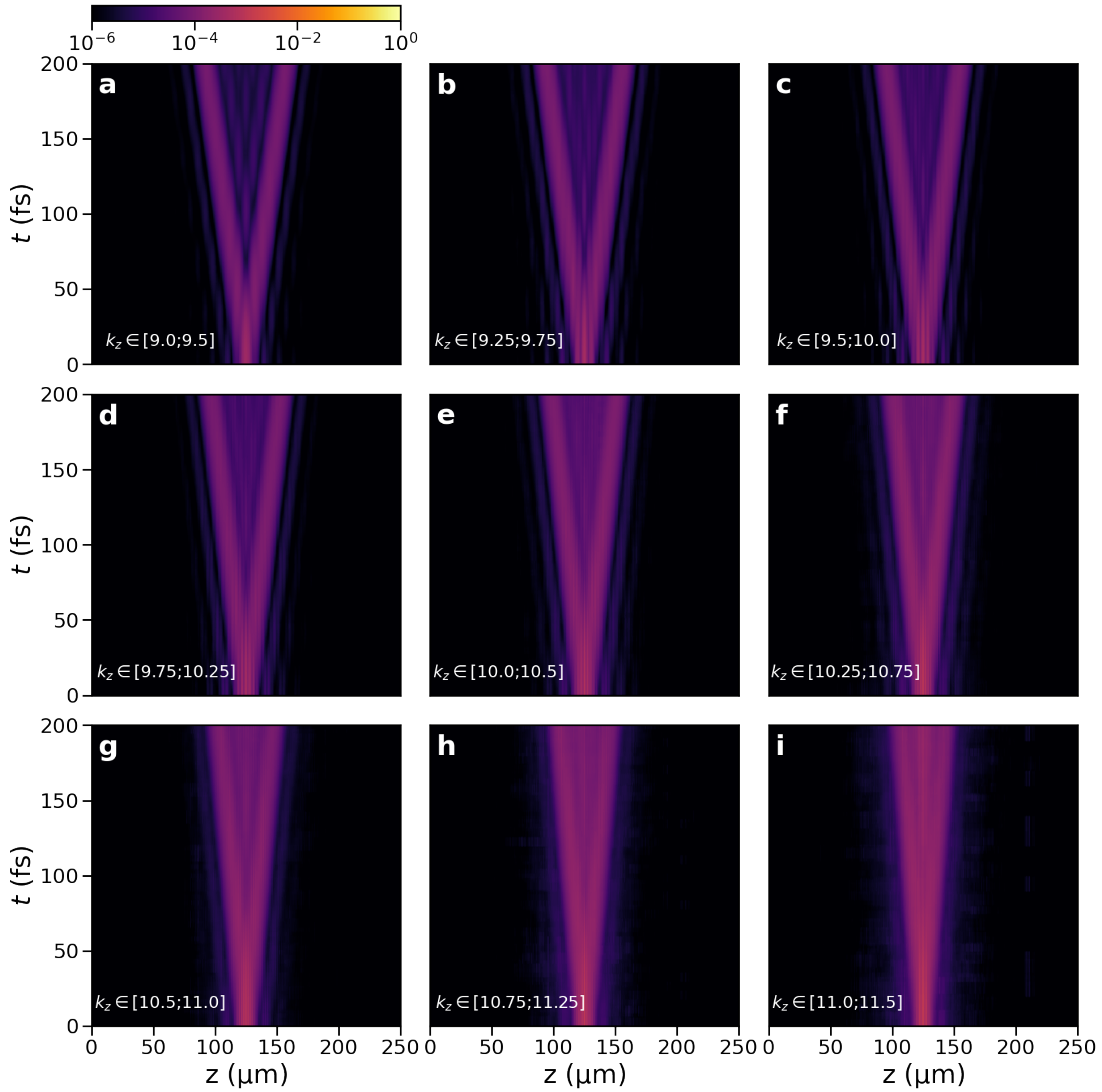

Figure S19: Time-space maps of the probability amplitude of the partial wave function, $|\Psi_w^{\text{part}}|^2$, associated with different energy and wave vector windows (Table S2) in quasi-dynamic simulations of two-level systems with the excitation energy disorder $\sigma = 63$ meV and average resampling time $\langle\tau\rangle = 10$ fs.

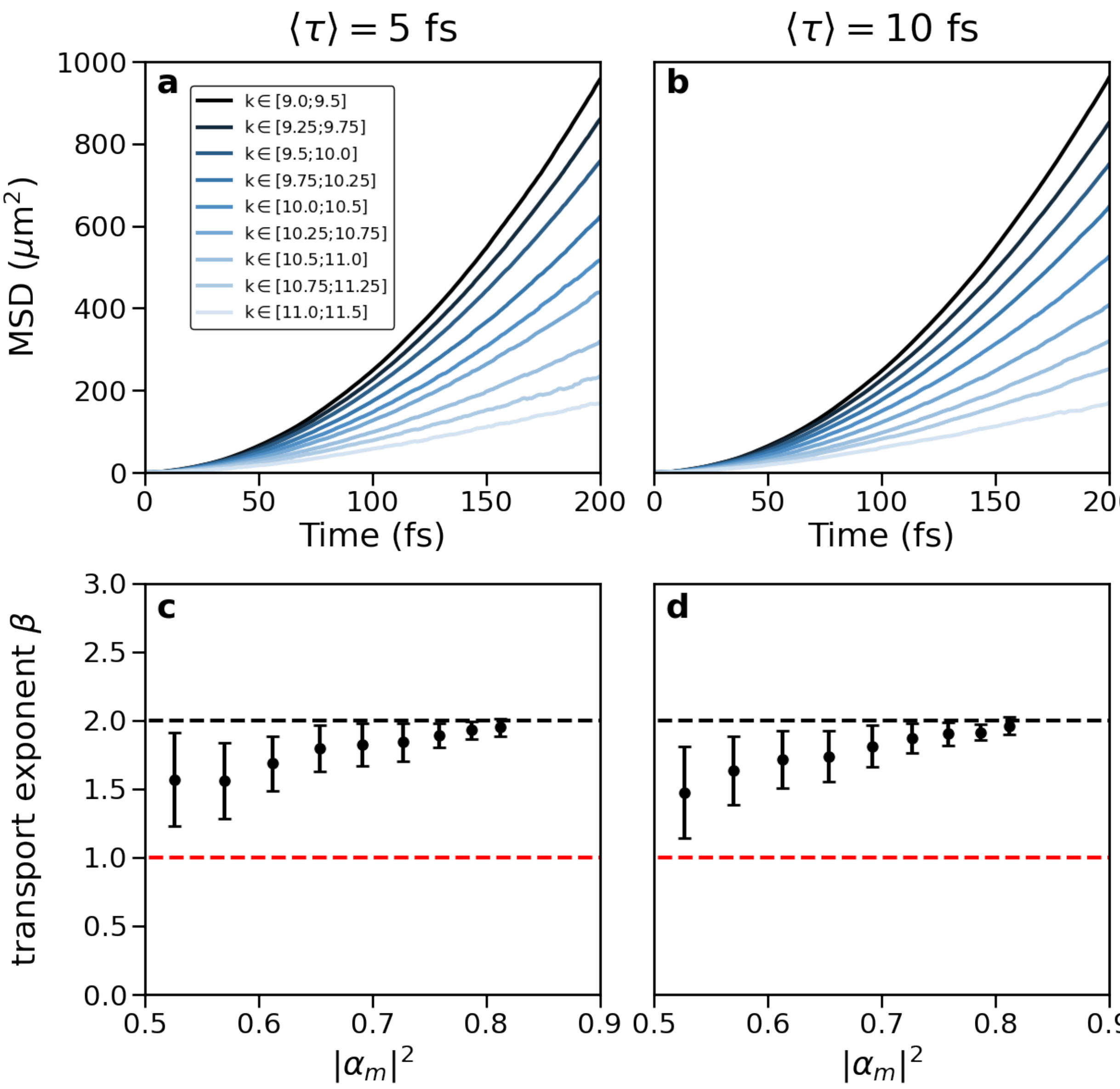

Figure S20: Panels **a** and **b**: Mean squared displacement (MSD_w) of the partial wave function $|\Psi^{\mathrm{part}}|^2$ extracted from different wave vector windows in quasi-dynamic simulations of two-level systems with the excitation energy disorder $\sigma = 63$ meV and the average resampling time $\langle\tau\rangle = 5$ fs (left panels) and $\langle\tau\rangle = 10$ fs (right panels). Panels **c** and **d**: Values of the transport exponent, β, as a function of the BSW modes contribution $|\alpha_\mathrm{m}|^2$ to polaritonic states extracted from the fit of the MSD_w to Equation 35. The errors are standard deviations of a hundred individual runs.

References

(1) Galego, J.; Garcia-Vidal, F. J.; Feist, J. Cavity-Induced Modifications of Molecular Structure in the Strong-Coupling Regime. *Phys. Rev. X* **2015**, *5*, 041022.

(2) Jaynes, E. T.; Cummings, F. W. Comparison of quantum and semiclassical radiation theories with application to the beam maser. *Proc. IEEE* **1963**, *51*, 89–109.

(3) Tavis, M.; Cummings, F. W. Approximate solutions for an N-molecule radiation-field Hamiltonian. *Phys. Rev.* **1969**, *188*, 692–695.

(4) Tichauer, R. H.; Feist, J.; Groenhof, G. Multi-scale Dynamics Simulations of Molecular Polaritons: the Effect of Multiple Cavity Modes on Polariton Relaxation. *J. Chem. Phys.* **2021**, *154*, 104112.

(5) Warshel, A.; Levitt, M. Theoretical studies of enzymatic reactions: Dielectric, electrostatic and steric stabilization of carbonium ion in the reaction of lysozyme. *J. Mol. Biol.* **1976**, *103*, 227–249.

(6) Boggio-Pasqua, M.; Burmeister, C. F.; Robb, M. A.; Groenhof, G. Photochemical reactions in biological systems: probing the effect of the environment by means of hybrid quantum chemistry/molecular mechanics simulations. *Phys. Chem. Chem. Phys.* **2012**, *14*, 7912–7928.

(7) Sakoda, K. *Optical Properties of Photonic Crystals*; Optical Sciences; Springer Berlin Heidelberg, 2005.

(8) Michetti, P.; Rocca, G. C. L. Polariton states in disordered organic microcavities. *Phys. Rev. B.* **2005**, *71*, 115320.

(9) Balasubrahmaniyam, M.; Simkovich, A.; Golombek, A.; Ankonina, G.; Schwartz, T. Unveiling the mixed nature of polaritonic transport: From enhanced diffusion to ballistic motion approaching the speed of light. *Nat. Mater.* **2023**, *22*, 338–344.

(10) Ehrenfest, P. Bemerkung über die angenäherte Gültigkeit der klassischen Mechanik innerhalb der Quantenmechanik. *Z. Phys.* **1927**, *45*, 445–457.

(11) Sokolovskii, I.; Goenhof, G. Non-Hermitian molecular dynamics simulations of exciton–polaritons in lossy cavities. *J. Chem. Phys.* **2024**, *160*, 092501.

(12) Lerario, G.; Ballarini, D.; Fieramosca, A.; Cannavale, A.; Genco, A.; Mangione, F.; Gambino, S.; Dominici, L.; Giorgi, M. D.; Gigli, G.; Sanvitto, D. High-speed flow of interacting organic polaritons. *Light Sci. Appl.* **2017**, *6*, e16212.

(13) Hou, S.; Khatoniar, M.; Ding, K.; Qu, Y.; Napolov, A.; Menon, V. M.; Forrest, S. R. Ultralong-Range Energy Transport in a Disordered Organic Semiconductor at Room Temperature Via Coherent Exciton-Polariton Propagation. *Adv. Mater.* **2020**, *32(28)*, 2002127.

(14) Rashidi, K.; Michail, E.; Salcido-Santacruz, B.; Paudel, Y.; Menon, V. M.; Sfeir, M. Y. Efficient and Tunable Photochemical Charge Transfer via Long-Lived Bloch Surface Wave Polaritons. *Arxiv* **2024**, *2409.02067v1*.

(15) Sokolovskii, I.; Tichauer, R. H.; Morozov, D.; Feist, J.; Groenhof, G. Multi-scale molecular dynamics simulations of enhanced energy transfer in organic molecules under strong coupling. *Nat. Commun.* **2023**, *14*, 6613.

(16) Jorgensen, W. L.; Chandrasekhar, J.; Madura, J. D.; Impey, R. W.; Klein, M. L. Comparison of simple potential functions for simulatin liquid water. *J. Chem. Phys.* **1983**, *79*, 926–935.

(17) Duan, Y.; Wu, C.; Chowdhury, S.; Lee, M. C.; Xiong, G. M.; Zhang, W.; Yang, R.; Cieplak, P.; Luo, R.; Lee, T.; Caldwell, J.; Wang, J. M.; Kollman, P. A point-charge force field for molecular mechanics simulations of proteins based on condensed-phase quantum mechanical calculations. *J. Comput. Chem.* **2003**, *24*, 1999–2012.

(18) Bayly, C. I.; Cieplak, P.; Cornell, W. D.; Kollman, P. A. A well-behaved electrostatic potential bsed method using charge restraints for deriving atomic charges - the RESP model. *J. Phys. Chem.* **1993**, *97*, 10269–10280.

(19) Tomasi, J.; Mennucci, B.; Cammi, R. Quantum Mechanical Continuum Solvation Models. *Chem. Rev.* **2005**, *105*, 2999–3094.

(20) Becke, A. D. A new mixing of Hartree-Fock and local density-functional theories. *J. Chem. Phys.* **1993**, *98*, 1372.

(21) Essmann, U.; Perera, L.; Berkowitz, M. L.; Darden, T.; Lee, H.; Pedersen, L. G. A smooth particle mesh Ewald potential. *J. Chem. Phys* **1995**, *103*, 8577–8592.

(22) Bussi, G.; Donadio, D.; Parrinello, M. Canonical sampling through velocity rescaling. *J. Chem. Phys.* **2007**, *126*, 014101.

(23) Berendsen, H.; Postma, J.; van Gunsteren, W.; la, A. D.; Haak, J. Molecular dynamics with coupling to an external bath. *J. Chem. Phys.* **1984**, *81*, 3684–3690.

(24) Miyamoto, S.; Kollman, P. A. SETTLE: An analytical version of the SHAKE and RATTLE algorithms for rigid water molecules. *J. Comp. Chem.* **1992**, *18*, 1463–1472.

(25) Becke, A. D. Density-functional thermochemistry. V. Systematic optimization of exchange-correlation functionals. *J. Chem. Phys.* **1997**, *107*, 8554–8560.

(26) Dunning, T. H. Basis Functions for Use in Molecular Calculations. I. Contractions of (9s5p) Atomic Basis Sets for the First-Row Atoms. *J. Chem. Phys.* **1970**, *53*, 2823–2833.

(27) Runge, E.; Gross, E. K. U. Density-Functional Theory for Time-Dependent Systems. *Phys. Rev. Lett.* **1984**, *52*, 997–1000.

(28) Hess, B.; Kutzner, C.; van der Spoel, D.; Lindahl, E. GROMACS 4: Algorithms for Highly Efficient, Load-Balanced, and Scalable Molecular Simulation. *J. Chem. Theory Comput.* **2008**, *4*, 435–447.

(29) Frisch, M. J.; Trucks, G. W.; Schlegel, H. B.; Scuseria, G. E.; Robb, M. A.; Cheeseman, J. R.; Scalmani, G.; Barone, V.; Petersson, G. A.; Nakatsuji, H.; Li, X.; Caricato, M.; Marenich, A. V.; Bloino, J.; Janesko, B. G.; Gomperts, R.; Mennucci, B.; Hratchian, H. P.; Ortiz, J. V.; Izmaylov, A. F.; Sonnenberg, J. L.; Williams-Young, D.; Ding, F.; Lipparini, F.; Egidi, F.; Goings, J.; Peng, B.; Petrone, A.; Henderson, T.; Ranasinghe, D.; Zakrzewski, V. G.; Gao, J.; Rega, N.; Zheng, G.; Liang, W.; Hada, M.; Ehara, M.; Toyota, K.; Fukuda, R.; Hasegawa, J.; Ishida, M.; Nakajima, T.; Honda, Y.; Kitao, O.; Nakai, H.; Vreven, T.; Throssell, K.; Montgomery, J. A., Jr.; Peralta, J. E.; Ogliaro, F.; Bearpark, M. J.; Heyd, J. J.; Brothers, E. N.; Kudin, K. N.; Staroverov, V. N.; Keith, T. A.; Kobayashi, R.; Normand, J.; Raghavachari, K.; Rendell, A. P.; Burant, J. C.; Iyengar, S. S.; Tomasi, J.; Cossi, M.; Millam, J. M.; Klene, M.; Adamo, C.; Cammi, R.; Ochterski, J. W.; Martin, R. L.; Morokuma, K.; Farkas, O.; Foresman, J. B.; Fox, D. J. Gaussian~16 Revision C.01. 2016; Gaussian Inc. Wallingford CT.

(30) Hobson, P. A.; Barnes, W. L.; Lidzey, D. G.; Gehring, G. A.; Whittaker, D. M.; Skolnick, M. S.; Walker, S. Strong exciton–photon coupling in a low-Q all-metal mirror microcavity. *Appl. Phys. Lett.* **2002**, *81*, 3519–3521.

(31) Wenus, J.; Ceccarelli, S.; Lidzey, D. G.; Tolmachev, A. I.; Slominskii, J. L.; Bricks, J. L. Optical strong coupling in microcavities containing J-aggregates absorbing in near-infrared spectral range. *Org. Electron.* **2007**, *8*, 120–126.

(32) Xu, D.; Mandal, A.; Baxter, J. M.; Cheng, S.-W.; Lee, I.; Su, H.; Liu, S.; Reichman, D. R.; Delor, M. Ultrafast imaging of coherent polariton propagation and interactions. *Nat. Commun.* **2023**, *14*, 3881.

(33) Houdré, R.; Stanley, R. P.; Ilegems, M. Vacuum-field Rabi splitting in the presence of inhomogeneous broadening: Resolution of a homogeneous linewidth in an inhomogeneously broadened system. *Phys. Rev. A* **1996**, *53*, 2711–2715.

(34) del Pino, J.; Feist, J.; Garcia-Vidal, F. J. Quantum Theory of Collective Strong Coupling of Molecular Vibrations with a Microcavity Mode. *New J. Phys.* **2015**, *17*, 053040.

(35) Eizner, E.; Martínez-Martínez, L. A.; Yuen-Shou, J.; Kéna-Cohen, S. Inverting Singlet and Triplet Excited States using Strong Light-Matter Coupling. *Sci. Adv.* **2019**, *5*, aax4482.

(36) Martínez-Martínez, L. A.; Eizner, E.; Kéna-Cohen, S.; Yuemn-Zhou, K. Triplet harvesting in the polaritonic regime: A variational polaron approach. *J. Chem. Phys.* **2019**, *151*, 054106.

(37) Hess, B.; Bekker, H.; Berendsen, H. J. C.; Fraaije, J. G. E. M. LINCS: A linear constraint solver for molecular simulations. *J. Comput. Chem.* **1997**, *18*, 1463–1472.

(38) Tichauer, R. H.; Morozov, D.; Sokolovskii, I.; Toppari, J. J.; Groenhof, G. Identifying Vibrations that Control Non-Adiabatic Relaxation of Polaritons in Strongly Coupled Molecule-Cavity Systems. *J. Phys. Chem. Lett.* **2022**, *13*, 6259–6267.

(39) Yarkony, D. R. Nonadiabatic Quantum Chemistry—Past, Present, and Future. *Chem. Rev.* **2012**, *112*, 481–498.

(40) Cui, B.; Sukharev, M.; Nitzan, A. Short-time particle motion in one and two-dimensional lattices with site disorder. *J. Chem. Phys.* **2023**, *158*, 164112.
